# Lichen planus pemphigoides with predominant mucous membrane involvement: a series of 12 patients and a literature review

**DOI:** 10.3389/fimmu.2024.1243566

**Published:** 2024-04-15

**Authors:** Loraine Combemale, Gérôme Bohelay, Ishaï-Yaacov Sitbon, Btisseme Ahouach, Marina Alexandre, Antoine Martin, Francis Pascal, Isaac Soued, Serge Doan, Florence Morin, Sabine Grootenboer-Mignot, Frédéric Caux, Catherine Prost-Squarcioni, Christelle Le Roux-Villet

**Affiliations:** ^1^ Dermatology Department, Referral Center for Autoimmune Blistering Diseases, Assistance Publique des Hôpitaux de Paris (APHP), Avicenne Hospital and Sorbonne Paris Nord University, Bobigny, France; ^2^ Dermatology Department, Saint Pierre-Brugmann and Queen Fabiola Children’s University Hospitals, Université Libre de Bruxelles, Brussels, Belgium; ^3^ Pathology Department, Assistance Publique des Hôpitaux de Paris (APHP), Avicenne Hospital and Sorbonne Paris Nord University, Bobigny, France; ^4^ Ear, Nose and Throat (ENT) Department, Assistance Publique des Hôpitaux de Paris (APHP), Avicenne Hospital and Sorbonne Paris Nord University, Bobigny, France; ^5^ Ophthalmology Department, Assistance Publique des Hôpitaux de Paris (APHP), Bichat Hospital, Paris University, Paris, France; ^6^ Immunology Department, Assistance Publique des Hôpitaux de Paris (APHP), Saint-Louis Hospital, Paris University, Paris, France; ^7^ Immunology Department, Assistance Publique des Hôpitaux de Paris (APHP), Bichat Hospital, Paris University, Paris, France; ^8^ Histology Department, Sorbonne Paris Nord University, Bobigny, France

**Keywords:** lichen planus pemphigoides, mucous membrane pemphigoid, oral lichen planus, bullous pemphigoid, autoimmune blistering disease, autoimmune blistering dermatosis

## Abstract

**Background:**

Lichen planus pemphigoides (LPP), an association between lichen planus and bullous pemphigoid lesions, is a rare subepithelial autoimmune bullous disease. Mucous membrane involvement has been reported previously; however, it has never been specifically studied.

**Methods:**

We report on 12 cases of LPP with predominant or exclusive mucous membrane involvement. The diagnosis of LPP was based on the presence of lichenoid infiltrates in histology and immune deposits in the basement membrane zone in direct immunofluorescence and/or immunoelectron microscopy. Our systematic review of the literature, performed according to the Preferred Reporting Items for Systematic Reviews and Meta-Analyses guidelines, highlights the clinical and immunological characteristics of LPP, with or without mucous membrane involvement.

**Results:**

Corticosteroids are the most frequently used treatment, with better outcomes in LPP with skin involvement alone than in that with mucous membrane involvement. Our results suggest that immunomodulators represent an alternative first-line treatment for patients with predominant mucous membrane involvement.

## 1 Introduction

First described clinically by Kaposi (1), lichen planus pemphigoides (LPP) is commonly associated with lichen planus (LP) and bullous pemphigoid (BP). In addition to clinical (lichenoid lesions and tense blisters) and histological (lichenoid changes and subepidermal split) findings, the gold standard for the diagnosis of LPP is the detection of autoantibody deposits along the dermal–epidermal junction (DEJ) using direct immunofluorescence (DIF) on perilesional skin biopsies, as first reported by Stingl et al. (2). Circulating autoantibodies targeting type XVII collagen (COL17; molecular weight 180 kDa) in the sera of patients with LPP were first reported by Cognat et al. (3). Using immunoblotting (IB), Ogg et al. (4) demonstrated that these circulating anti-COL17 autoantibodies react with the membrane-proximal NC16A subdomain.

Additional studies suggested that LPP is not a simple association between LP and BP and highlighted its heterogeneity regarding targeted antigens. The primary antigenic target of LPP is COL17; however, other antigenic targets have also been identified. Zillikens et al. (5) showed that a novel epitope within the BP-NC16A domain, designated as MCW-4, is recognized by the serum autoantibodies in patients with LPP. Moreover, using recombinant proteins covering the entire NC16 domain of BP-180, the same team identified more subtle differences in the epitope specificity of circulating autoantibodies in the sera of patients with LPP compared with those with mucous membrane pemphigoid (MMP), BP, and pemphigoid gestationis (PG) (6). Other targets of autoantibodies described in LPP are the C-terminal domains of BP180 (7–10), BP230, LAD antigen (8, 11), desmoglein 1 (7, 8), and an antigen with a molecular weight of 200 kDa (12, 13).

Clinically, LPP is often described as the development of blisters on the skin, in not only areas with lichenoid changes but also uninvolved areas. Mucous membrane (MM) involvement has mainly been observed in the oral mucosa. Indeed, Zaraa et al. (14), in their review of literature published between 1980 and 2010, reported oral involvement in 28 of 78 (36%) cases, but rarely in other MM, and did not report LPP with predominant mucosal involvement.

Herein, we report 12 cases of LPP with exclusive or predominant MM involvement diagnosed at our center. To date, this is the largest reported study of LPP with predominant MM involvement. We described the clinical, histological, and immunological characteristics of these LPP cases and compared our findings with those of previous studies on LPP with and without MM involvement via a systematic review, according to the Preferred Reporting Items for Systematic Reviews and Meta-Analyses (PRISMA) guidelines.

## 2 Patients and methods

This single-center, retrospective study was conducted in May 2022 and involved patients treated between 2001 and 2020 at Avicenne Hospital (Assistance Publique-Hôpitaux de Paris, Bobigny, France) using the computer database (eDBAI) of the referral center for autoimmune bullous diseases (AIBD). The information of all patients was systematically recorded and stored in a computerized medical chart standardized for AIBD after obtaining written informed consent from the patients. This study was approved by the institutional review board of Avicenne Hospital (approval number: CLEA-2022-240).

### 2.1 Patient selection

Among patients with AIBD monitored at our center, those with a confirmed diagnosis of LPP were included. The inclusion criteria were a diagnosis of (i) LP relying on clinical data (white network lesions of MM and/or pruritic violaceous papules on the skin) with histological confirmation (lichenoid interface dermatitis) and (ii) sub-epithelial/epidermal AIBD relying on clinical data (blisters), histological data (subepithelial/epidermal cleavage), and autoantibody deposits (IgA, IgG, and IgM) along the DEJ or chorioepithelial junction (CEJ) detected using DIF and/or direct immunoelectron microscopy (IEM).

### 2.2 Data collection

The patients were designated with numbers N°1–12 (**Table 1**), and the following data were collected from each patient’s medical record:

- Epidemiological characteristics, including sex, age, comorbidities, medications, and LPP diagnosis time.
- Clinical findings on the physical examination of LP papules, LP MM white network, blisters, erosions, erythema, atrophy, and synechiae, as well as their locations on the MM and/or skin.
- Histological and immunological findings of skin and/or MM biopsies at diagnosis, including lichenoid interface dermatitis (band-like lymphocytic infiltrate, degeneration of basal keratinocytes, with varying degrees of epithelial lymphocytic exocytosis and necrosis of basal keratinocytes) and subepithelial/epidermal cleavage, as well as Ig class(es) (IgA, IgG, and IgM), ± C3 deposits at the CEJ, and/or DEJ on DIF, and/or direct IEM (on semi-thin sections), and ultrastructural immune deposits’ locations on direct IEM (on ultrathin sections).
- Immunoserology results at diagnosis, including indirect immunofluorescence (IIF) with patient sera on rat and/or monkey oesophagus (BMD, Marne la Vallée, France, The Binding Site, Saint Egrève, France); IIF on 1 M NaCl-treated human or monkey salt-split skin (IIF-SSS) (Immco Diagnostics, Buffalo, USA); commercially available BP180-NC16A, BP230, and type 7 collagen enzyme-linked immunosorbent assays (ELISAs) (MBL, Nagoya, Japan); and immunoblotting (IB) using the human amniotic membrane (15).
- Treatments, including topical steroids, dapsone, sulfasalazine, doxycycline, acitretin, systemic corticosteroids, and immunosuppressants (mycophenolate mofetil and rituximab).
- Follow-up and status at the last visit, including complete remission (CR), almost CR (aCR; transient new lesions that heal within 1 week), controlled disease, or active disease adapted from the consensus statement for MMP (16). The active lesions included erythema, erosion, and blisters. A persistent mucosal residual lichenoid network without inflammation was not considered active disease.

**Table 1:** Clinical data of our patients with lichen planus pemphigoid predominant on mucous membrane.

Patient no.	Sex	Age (years)	Disease duration at LPP diagnosis (months)	Order of diagnosis between LP and AIBD	Sites involved at LPP diagnosis			Clinical data				
					Skin yes/no	MM n	MM sites	Cutaneous LP papules	MM LP network	Cutaneous blisters	MM blisters	Other lesions
1	F	86	15	LP first	yes	2	B, G	yes	no	yes	yes	atrophia, synechiae
2	F	43	18	LP first	yes	2	B, NT	yes	no	no	yes	atrophia
3	M	58	24	LP first	yes	3	B, G, A	yes	yes	no	yes	erosion
4	F	74	96	LP first	yes	2	B, G	no	yes	yes	yes	atrophia, erosion, synechiae
5	F	82	24	LP first	yes	3	B, G, NT	no	yes	yes	yes	atrophia, erosion, synechiae
6	M	69	156	LP first	no	3	B, G, A	no	yes	no	no	erosion
7	F	71	36	LP first	no	2	B, NT	no	yes	no	no	atrophia, erosion
8	M	35	228	LP first	no	2	B, G	no	yes	no	yes	atrophia, synechiae
9	F	58	8	AIBD first	yes	2	B, NT	no	yes	yes	yes	synechiae
10	F	65	48	AIBD first	no	2	G, C	no	no	no	no	synechiae
11	F	42	4	at once	yes	2	B, G	no	yes	yes	yes	erosion
12	F	66	nd	at once	yes	3	B, G, A	yes	yes	yes	no	atrophia, erosion,

LPP, Lichen Planus Pemphigoid; LP, Lichen Planus; AIBD, autoimmune bullous disease; n, number; MM, mucous membrane; F, female; B, buccal mucous membrane; G, genitalia; NT, nose and throat; M, male; A, anal; C, conjunctiva; nd, not determinated.

### 2.3 Literature review

The medical literature search was performed following the PRISMA guidelines (17). Bibliographical research was carried out using the PubMed database for the period between 1975 and May 2022 using the following keywords: “lichen planus” AND “pemphigoid.” Cases of histologically proven LP and positive DIF, case reports and series, and manuscripts written in English or French were included in this systematic literature review.

### 2.4 Statistical analysis

Statistical analyses were performed using StatView (v5.0, SAS Institute Inc.). Quantitative variables are expressed as the medians and interquartile ranges or extreme values, as indicated, according to normality assessed using the Shapiro–Wilk test. Qualitative variables are presented as numbers and proportions. Quantitative variables were analyzed via univariate comparisons between subgroups using Mann–Whitney tests; qualitative variables were analyzed via univariate comparisons using Pearson’s χ^2^ tests, with or without Yate’s continuity correction, or Fisher’s exact tests, as appropriate, according to sample size.

## 3 Results

### 3.1 Patients and clinical findings

In total, 12 patients (nine females and three males, ratio 3:1), with a median age of 65.5 years (range: 35–86 years), were included in this study (**Table 1**). No patient had a medical history of other autoimmune diseases or chronic infections, such as diabetes or hepatitis. Two patients had a medical history of lymphoma: one had Hodgkin lymphoma a few years before LPP (N°3), and the other was diagnosed with LPP during the course of non-Hodgkin lymphoma therapy (N°11). The latter was the only patient among the 12 (8.3%) to have a plausible drug-induced LPP—the culprit drug being pembrolizumab.

The median time between the first symptoms and diagnosis of LPP was 5.4 years (0.7–19.0 years). In eight patients (66%; N°1–8), the lichenoid component preceded the first evidence of autoimmune blistering components by 1.2–13.0 years (mean value: 4.7 years). In two patients (16.7%; N°9–10), typical blistering lesions involving MM preceded (clinically and histologically) typical lichenoid lesions (N°9–10). In the last two patients (N°11–12), LPP was suspected from the outset because of both typical LP lesions and blisters on the MM.

**Figure 1:**
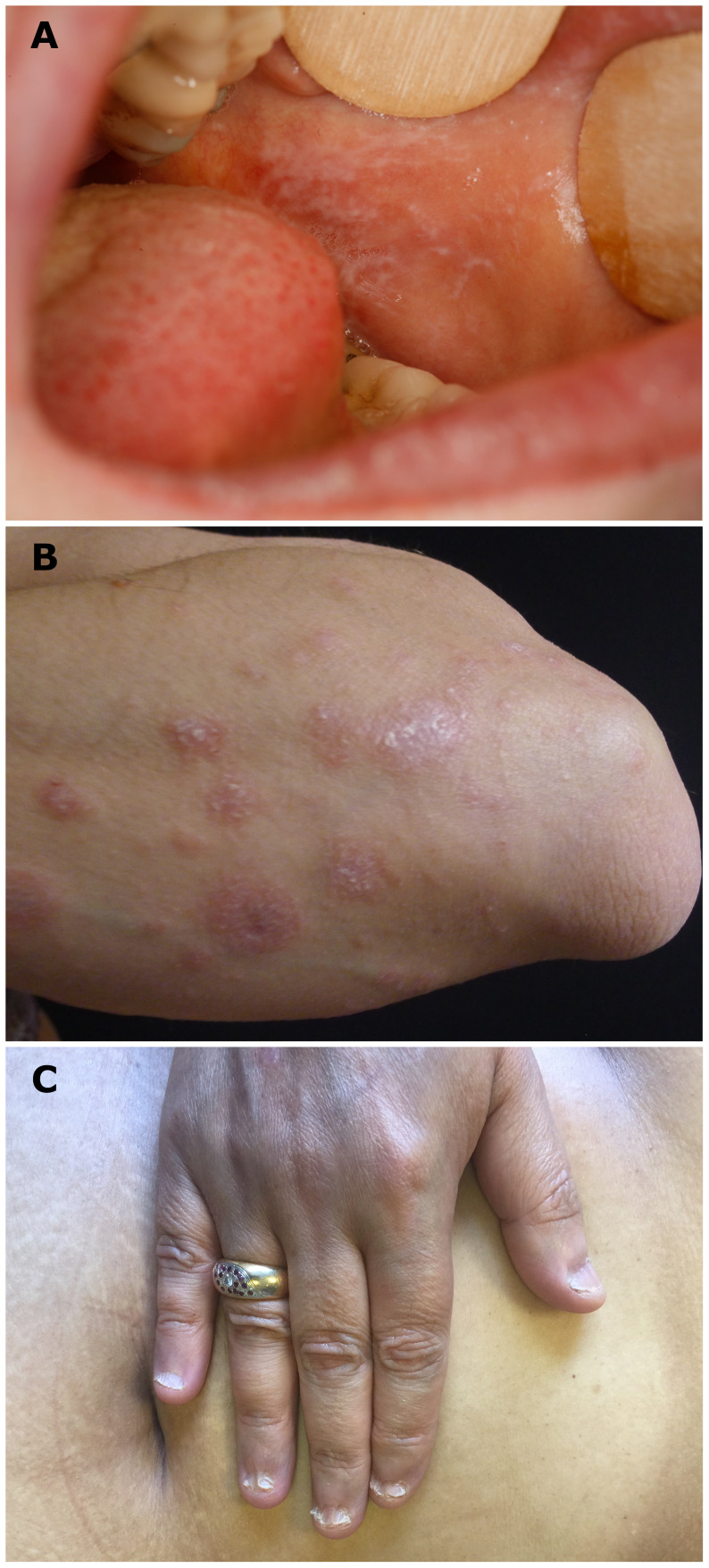
Typical clinical lichenoid lesions. **(A)** White reticulated network on buccal mucosa (patient N°9), **(B)** cutaneous violaceous papules with Wickham network on a left forearm (patient N°2), and **(C)** lichen planus onychodystrophy (pterygium) of one patient (patient N°12).

**Figure 2:**
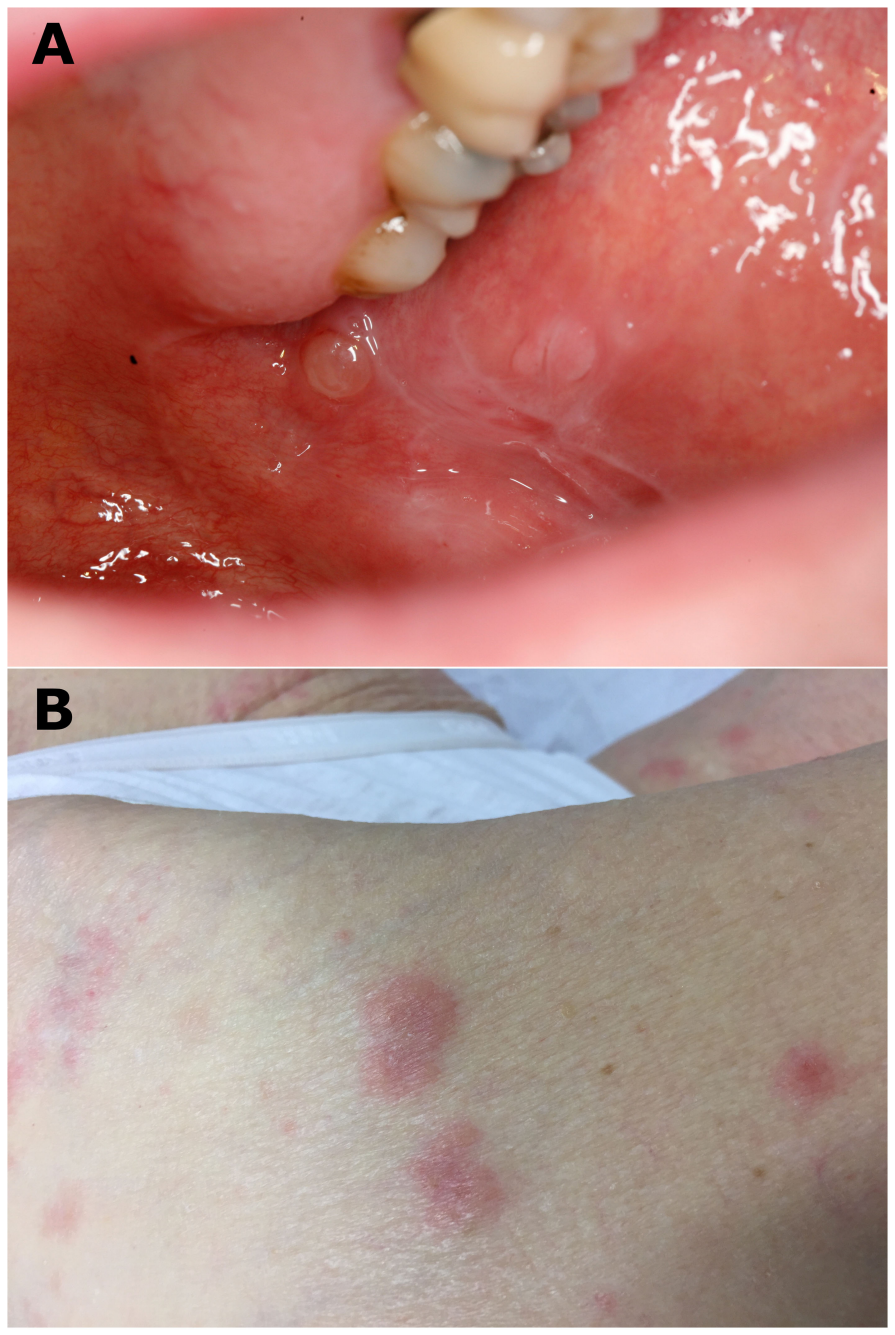
Typical clinical bullous lesions. **(A)** Blisters and white reticulated network observed on buccal mucosa (patient N°3) and **(B)** bullous and erythematous lesions on a leg (patient N°4).

**Figure 3:**
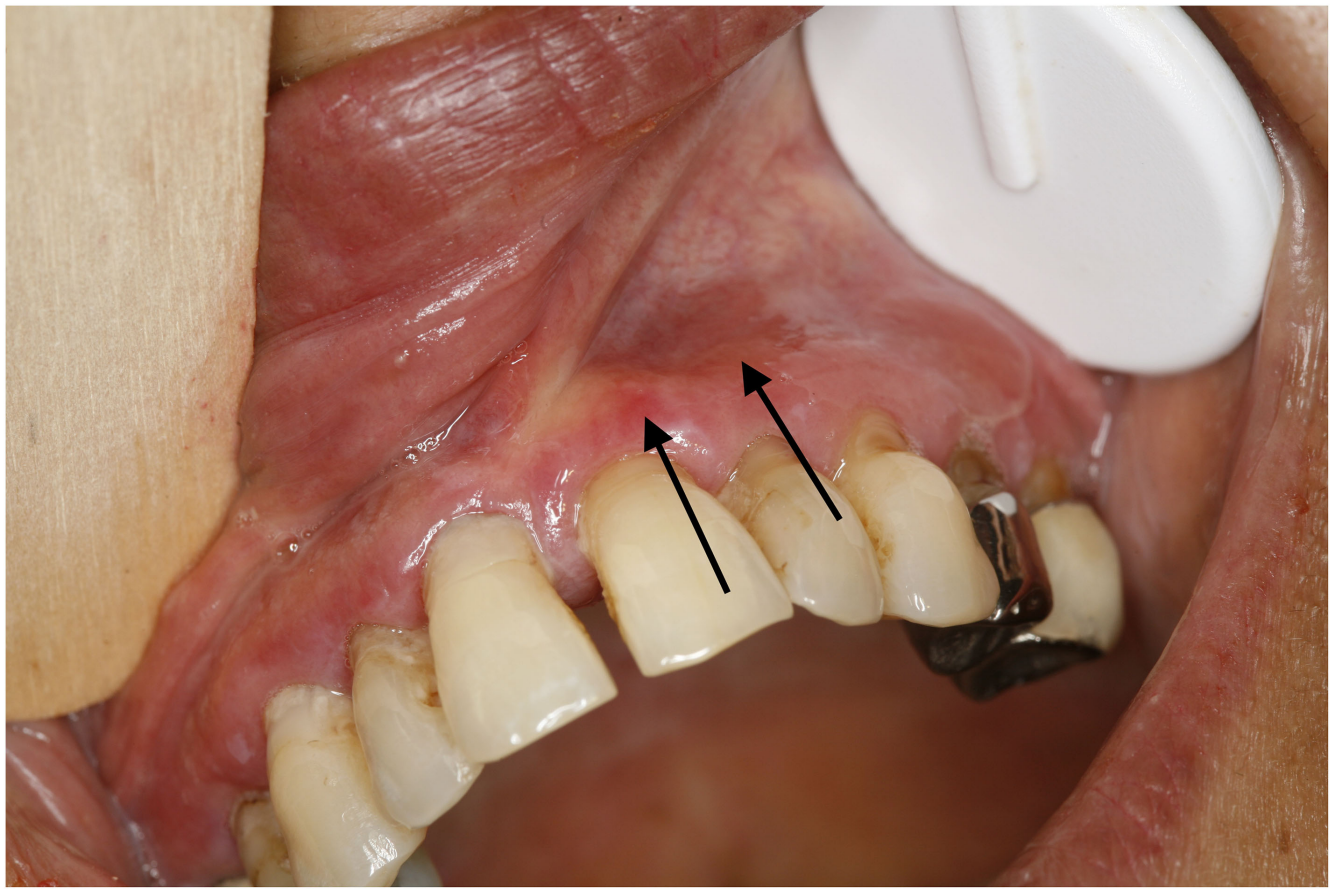
Erythema (black arrows), erosion, white lichenoid lesion, and atrophy on vestibular gingiva. Erythema could be a manifestation of lichen planus or mucous membrane pemphigoid. (patient N°12).

Upon clinical examination, all patients had mucosal involvement affecting one to three different MMs. The MMs involved were the oral (11 of 12), genital (nine of 12), nose and throat (NT; four of 12), anal (three of 12), and conjunctival (one of 12) mucosa; skin lesions were associated in eight of these (66%). Among the 12 patients, four had exclusive MM involvement; the remaining eight had predominant MM involvement, with at least two MM involvements. Moreover, 11 patients had typical LP lesions involving the skin and/or the MM (**Figure 1**). A white reticulated network in the oral and anal MM (**Figure 1A**) was observed in nine (N°3–9, 11, 12) and one (N°3) of the 12 patients, respectively. Cutaneous violaceous papules with a Wickham network (**Figure 1B**) were observed in four patients (N°1–3, 12), associated with nail involvement (pterygium; **Figure 1C**) in one patient (N°12). One patient (N°10) had typical characteristics of LP on histological examination, without typical clinical lesions of the lichen (see below). Nine patients (N°1–5, 8–9, 11–12) had blisters on the MM and/or skin, eight on the oral MM, and six on the skin (**Figure 2**). In addition to typical LP and autoimmune blistering disease (AIBD) lesions, all patients demonstrated non-specific clinical lesions that could be found in both LP and AIBD (erythema, erosion, atrophy, synechiae, and conjunctival fibrosis) on their MM [oral, eight of 12; genital, eight of 12; NT, two of 12; anal, two of 12; and/or conjunctival, one of 12, patient N°12, fibrosis of stage IIIA in Tauber and Foster’s classification (18); **Figure 3**].

### 3.2 Histological and immunological data of skin and MM biopsies

**Figure 4:**
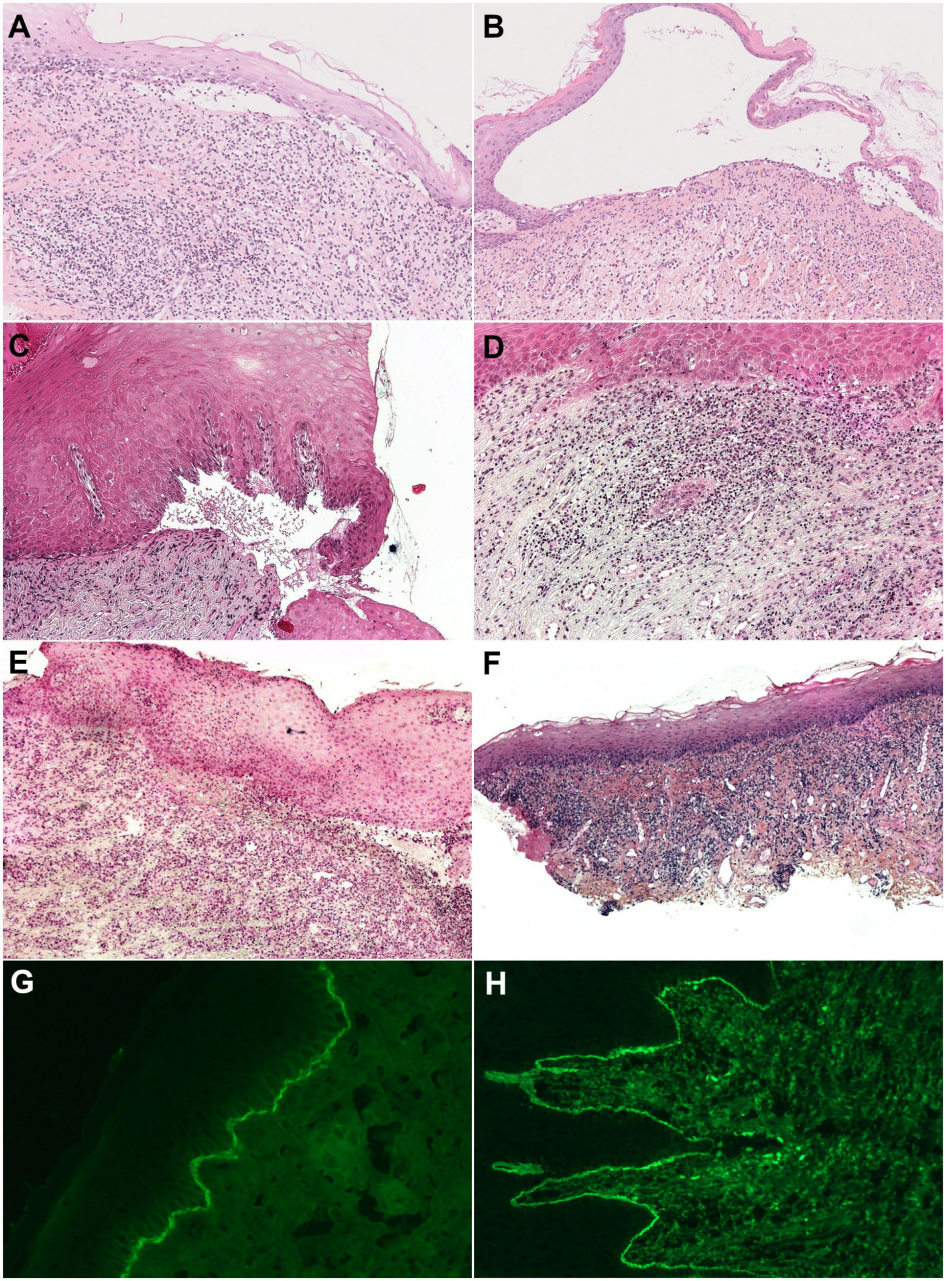
Histology of mucous membrane biopsy (Hematoxylin and eosin staining) and subepithelial cleavage and lichenoid reaction pattern (band-like lymphocytic infiltrate hugging the chorioepithelial junction with, depending on the patient, vacuolar degeneration of the basal layer of the epithelium, necrosis of individual keratinocytes, and lymphocytic exocytosis). **(A, B)** Both in one section of a biopsy (original magnification, 50×), with **(A)** discrete subepithelial cleavage and predominant lichenoid reaction (patient N° 8) and **(B)** large subepithelial cleavage and discrete lichenoid pattern with lymphocytic exocytosis (patient N° 12). **(C, D)** Separately in two different sections of one biopsy (original magnification, 100×), with **(C)** subepithelial cleavage and **(D)** a lichenoid reaction pattern (patient N°3). **(E, F)** Separately in two different biopsies (original magnification, 100×), with **(E)** subepithelial cleavage on gingiva biopsy and **(F)** a lichenoid reaction pattern on buccal mucosa biopsy (patient N°5). **(G, H)** Direct immunofluorescence, with **(G)** linear immune deposits of IgG on the DEJ (patient N°11) and **(H)** on the CEJ (patient N°4).

Each patient underwent an average of two biopsies, including at least one mucosal biopsy, to confirm the LPP diagnosis. Most patients required separate biopsies of lesions suggestive of LP and MMP, with multiple biopsies necessary in some. Subepithelial/epidermal cleavage was observed in eight of the 12 patients (**Table 2**). All patients had the histological characteristics of LP, including subepidermal band-like lymphocytic infiltrate in 11 patients, necrotic keratinocytes in seven patients, and lymphocytic exocytosis in eight patients (**Figure 4**). Subepithelial cleavage and lichenoid reaction patterns could be observed in one section (**Figures 4A, B**), separately in two different sections of a unique biopsy sample (**Figures 4C, D**), or separately in two samples obtained from two different sites (**Figures 4E, F**).

**Table 2:** Histological and immunological data of our patients with lichen planus pemphigoid predominant on mucous membrane.

Patient no.	Lichenoid typical features	Subepidermal band-like lymphocytic infiltrate	Necrotic keratinocytes	Lymphocytic exocytosis	Sub-epithelial cleavage	DIF: BMZ (IgG, IgA, IgM, C3)	DIEM (SF): BMZ	DIEM (UF): HD,LL, LD	IIF/ IIF-SSS: BMZ/ roof, floor	ELISAs: BP180, BP230 COL VII	WB (amniotic extracts)
1	yes	yes	yes	no	yes	+ (G, C3)	+	LD, LL	neg	BP180 +/neg/nd	nd
2	yes	no	yes	no	yes	+ (G, A M, C3)	+	HD, LL	neg	neg/neg/neg	nd
3	yes	yes	yes	yes	yes	neg	+	LD, LL	neg	neg/neg/nd	nd
4	yes	yes	no	yes	no	+ (G, C3)	+	HD, LL	+/roof	BP180 +/neg/nd	nd
5	yes	yes	yes	yes	yes	+ (G, A)	+	LD	neg	BP180 +/neg/neg	neg
6	yes	yes	yes	yes	no	neg	+	LD, LL	neg	neg/neg/neg	nd
7	yes	yes	no	no	yes	neg	+	nd	neg	neg/neg/nd	nd
8	yes	yes	yes	no	yes	neg	+	HD	neg	BP180 +/BP230 +/Col VII +	nd
9	yes	yes	no	yes	no	+ (G, C3)	+	LD, LL	+/roof	neg/neg/nd	neg
10	yes	yes	no	yes	no	+ (C3)	nd	nd	neg	neg/neg/neg	200kDa band
11	yes	yes	yes	yes	yes	+ (G, C3)	+	nd	neg	BP180 +/neg/neg	nd
12	yes	yes	no	yes	yes	+ (G, C3)	+	HD, LL	+/roof	BP180 +/BP230 +/nd	nd

DIF, direct immunofluorescence; BMZ, basal membrane zone; Ig, immunoglobulin; C3, complement C3; DIEM, direct immunoelectron microscopy; SF, semi-thin section; UF, ultra-thin section; HD, hemidesmosome; LL, lamina lucida; LD, lamina densa; IIF, indirect immunofluorescence; IIF-SSS, immunofluorescence on salt-split skin; ELISA, enzyme-linked immunosorbent assay; BP, bullous pemphigoid antigen; col VII, type VII collagen; WB, Western blot assay; +, positive; neg, negative; nd, not determinated.

**Figure 5:**
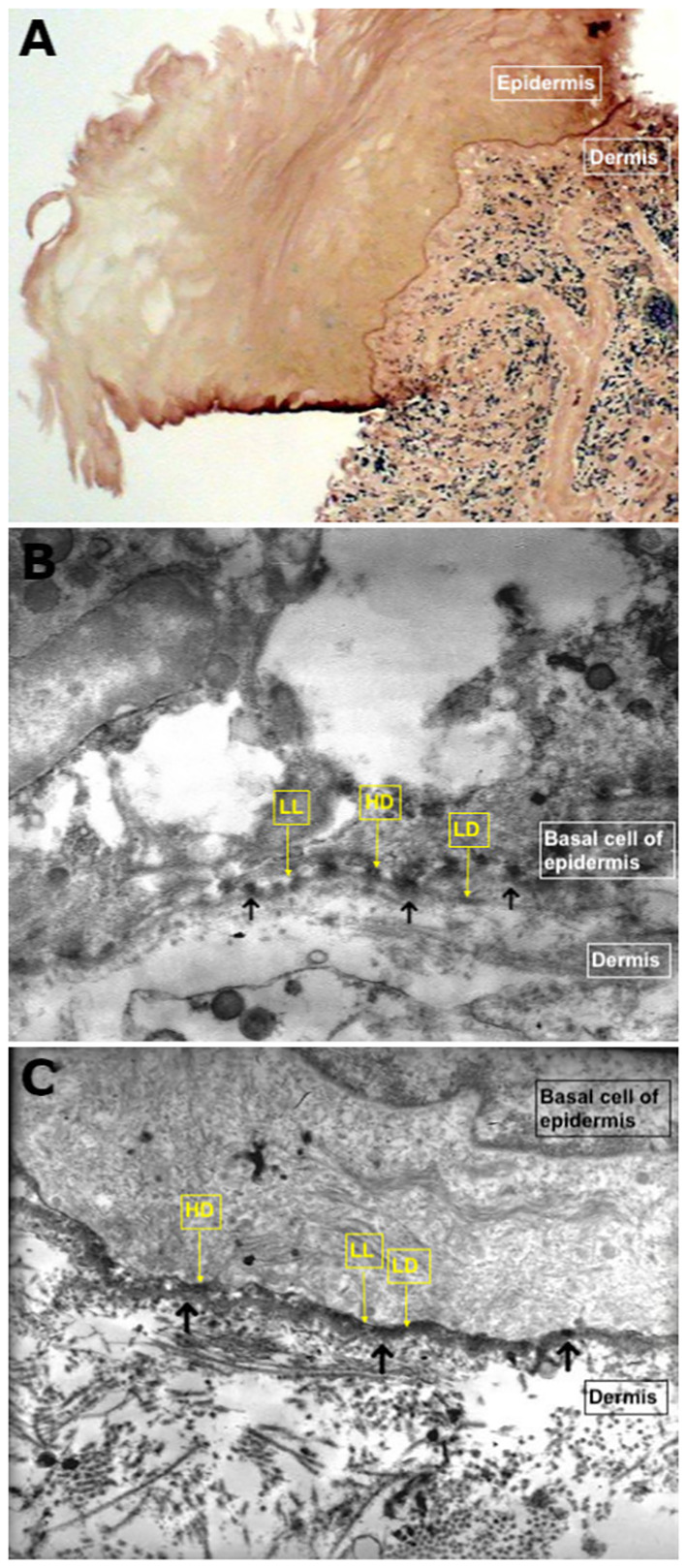
Direct immunoelectron microscopy. **(A)** Semi-thin section demonstrating linear deposits of autoantibodies on the chorioepithelial junction (original magnification, 40×; patient N°2). **(B)** Ultra-thin section demonstrating autoimmune deposits on hemidesmosomes or hemidesmosomes and lamina lucida (shown by the black arrows) (original magnification, 15,000×; patient N°4). **(C)** Ultra-thin section demonstrating thick immune deposits (shown by the black arrows) on lamina densa intermittently overlying the lamina lucida (original magnification, 15,000×; patient N°1).

DIF and/or direct IEM of semi-thin sections (**Figures 4G, H**, **5A**) revealed linear deposits of autoantibodies (IgG and IgA) on JDE/JCE in all patients (**Table 2**). IEM on ultrathin sections performed in nine of the 11 patients exhibited autoantibody deposits (IgG and IgA) in all samples studied, of which four had a negative DIF. IgG and/or IgA deposits were located on hemidesmosomes (HD) or on HD and lamina lucida (LL) in four of the 11 patients or on the lamina densa (LD) or LD and LL in five of the 11 patients (**Figures 5B, C**). It was non-contributory in two of the 11 patients.

### 3.3 Circulating autoantibodies

Standard IIF on rat/monkey oesophagus and IIF-SSS were positive in the sera of three of 12 patients (N° 4, 9, 12), with labeling of the epidermal side on SSS (**Table 2**).

ELISA demonstrated the presence of circulating IgG autoantibodies directed against BP180-NC16A in six patients (50%; N° 1,4,5,8,11,12), BP230 in two patients (N° 8,12), and type VII collagen in one patient (N° 8) (**Table 2**). IB using amniotic membrane extracts was performed only on the sera of three patients; the serum of one patient (N°10) showed IgG autoantibodies with a 200-kDa band (**Table 2**).

### 3.4 Treatment and follow-up

All 12 patients received topical corticosteroids, notably awaiting confirmation of LPP diagnosis (**Table 3**). All but one patient received systemic treatment. Systemic therapies used in the first-line treatment mostly comprised immunomodulatory drugs (11 of 12), such as dapsone, doxycycline, and sulfasalazine, in combination with systemic corticosteroids for one patient. Acitretin was also administered to treat the LP component (three of 12 patients) as the first- or second-line treatment when LP lesions seemed particularly active. Dapsone was administered as a first-line treatment to nine of the 12 patients; six of the 12 patients received dapsone alone and three of the eight patients received it in combination with other drugs (systemic corticosteroids [N°5], sulfasalazine [N°11], and doxycycline and acitretin [N°12]). Six patients were in CR or aCR after first-line treatment, of whom five were treated with dapsone alone (N°3, 4, 6, 9, 10). The other patient was treated with dapsone combined with doxycycline (200 mg) and acitretin (0.3–0.5 mg/kg) (**Table 3**).

**Table 3:** Treatment and outcome data of our patients with lichen planus pemphigoid predominant on mucous membrane.

Patient	Treatment (maximum dose)		Clinical status (last visit)	Follow-up (years)	Relapses (number) and type of lesions	
no.	First-line	Second-line			Lichenoid	AIBD
**1**	DOX 200 mg	DDS 1 mg/kg	CR	5	0	3
**2**	DDS 1.3 mg/kg	DDS 1.3 mg/kg + MMF 3.5g + ACT 0.3 mg/kg	aCR	9	2	no CR
**3**	DDS 0.5 mg/kg		CR	6	0	0
**4**	DDS 2 mg/kg		aCR	6	6	3
**5**	DDS 0.6 mg/kg + sCS 0.3 mg/kg	DDS 0.6 mg/kg + sCS 0.3 mg/kg + RTX	AD	4	1	1
**6**	DDS 2.6 mg/kg	tCS	AD	7	0	1
**7**	tCS mouth wash		CR	2	2	0
**8**	DOX 200mg	DDS 1mg/kg + SSZ 1g	controlled	2	6	4
**9**	DDS 1 mg/kg		CR	9	1	1
**10**	DDS 1 mg/kg		CR	5	0	0
**11**	DDS 2 mg/kg + SSZ 1g	DDS 2 mg/kg + SSZ 1g + ACT 0,1 mg/kg + RTX 2 g	AD	2	0	no CR
**12**	DDS 2 mg/kg + DOX 200 mg, + ACT 0.3-0.5 mg/kg		aCR	19	10	2

AIBD, autoimmune bullous disease; DOX, Doxycycline; DDS, Dapsone; CR, Complete Remission; MMF, Mycophenolate Mofetil; ACT, Acitretin; aCR, Almost Complete Remission; sCS, Systemic Corticosteroids; RTX, Rituximab; AD, Active Disease; tCS, Topical Corticosteroids; SSZ, Sulfasalazine.

Among the 12 patients, five received more than one line of treatment (**Table 3**). The molecules administered as second-line treatments were immunomodulators, systemic corticosteroids, and/or immunosuppressants (mycophenolate mofetil or rituximab). All the patients received dapsone therapy. Two of the five patients achieved CR or aCR (N°1, 2), one had controlled disease (N°8), and the last two still presented with active disease (N°5, 11) at the last follow-up. One patient (N°6) in the CR group developed transient aplasia, and dapsone was discontinued.

Relapse occurred in 10 of the 12 patients, most of whom had lichenoid lesions; three of the 12 patients had frequent flares. Notably, relapse of bullous lesions occurred in four of the seven patients because of the decrease or discontinuation of immunomodulators.

At the last visit, after a median follow up of 5.5 years (range: 2–19 years), eight patients (66.7%) were in CR or aCR, one had a controlled disease, and three still had active disease (**Table 3**).

## 4 Literature review

Only 132 patients with LPP have been reported in 112 case reports or small series since the first description of autoimmune deposits on the basement membrane zone (BMZ) in LPP, 50 years ago by Sting et al. (2) (**Annex 1**). A predominance of females (60%, 80 females vs. 52 males) was observed, with a median age at diagnosis of 48.9 years, and 19 paediatric cases of LPP have been reported (14.4%). Comorbidities included diabetes in 17 patients (12%), chronic viral hepatitis in 4 (3%), and cancer in 23 (16%). LPP was considered as drug induced in 24 cases (17%), the suspect drugs mostly being anti-PD1 or anti-PDL1.

Among the reported cases, 71 patients (54%) had mucosal involvement (**Table 4**). Most of these patients were initially diagnosed with LP (75.4%); only a few cases were diagnosed with LPP at the outset (21.5%). Clinically, oral mucosa is the most frequent site of involvement (97%), followed by skin (82%), genitals (18.3%), eyes, and ENT (both 4.2%); one case of oesophageal involvement has been described (19). Blisters (77.5%) and lichenoid lesions (80.3%) were mostly observed on the skin. A total of 56 patients (78.9%) presented histological features of subepidermal AIBD. Several biopsies were required to diagnose LPP (mean number of 1.7 biopsies per patient). All DIF cases were positive, and cases without positive results were excluded from the literature review. Circulating autoantibodies were found in 83.7% of patients; IIF/IIF-SSS was positive in 73.9% of cases, labeling most frequently the roof (77.3%), as opposed to the floor (4.5%). Autoantibodies reacted more frequently with BP180-NC16A (88.5%) than with BP230 (26.7%) on ELISA. IB also detected anti-BP180 autoantibodies more frequently than anti-BP230 antibodies. In 10 patients in whom IB was specified, antibodies were directed against NC16A (six of 10) and the C-terminal region of BP180 (two of 10). Regarding treatment, corticosteroids were widely used as the first-line treatment (48.6% alone and 14.3% combined), immunosuppressive and immunomodulatory drugs were used in 11.4% and 8.6% of patients, respectively. Remission (CR and aCR) was achieved in 65% of cases at the end of follow up (median, 10.5 months). Subsequent lines of treatment included combined therapies, immunomodulators, and retinoids, and one patient also received rituximab.

**Table 4:** Comparison between our series and the MM-LPP cases from the literature.

Variables	All cases N =83	MM-LPP our series N = 12	MM-LPP litterature N = 71	p-value
Female gender, N (%)	56 (67.5)	9 (75.0)	47 (66.2)	0.7426
Age at diagnosis (years), median (IQR)	58 (23.0)	65.5 (22.0)	57 (21.5)	0.0922
Diabetes (%)	9 (10.8)	0 (0)	9 (12.7)	0.3445
Chronic viral hepatitis (%)	3 (3.6)	0 (0)	3 (3.0)	>.9999
Cancer (%)	15 (18.1)	2 (16.7)	13 (18.3)	>.9999
Drug induction (%)	10 (12.0)	1 (8.3)	9 (12.7)	>.9999
Order of diagnosis (%) – available for n=	74 (89.2)	12 (100)	62 (87.3)	
LP first	57 (74.0)	8 (66.7)	49 (75.4)	0.4977
AIBD first	4 (5.2)	2 (16.7)	2 (3.1)	0.1124
At once	16 (20.8)	2 (16.7)	14 (21.5)	>.9999
Clinical presentation (%)				
Mouth	80 (96.4)	11 (91.7)	69 (97.2)	0.3779
Skin	66 (79.5)	8 (66.7)	58 (81.7)	0.2553
**Genital**	**22 (26.5)**	**9** (75.0)	**13** (18.3)	**0.0002**
Eyes	4 (4.8)	1 (8.3)	3 (4.2)	0.4713
**NT**	**7 (8.4)**	**4 (33.3)**	**3 (4.2)**	**0.0073**
Oesophagus	1 (1.2)	0 (0)	1 (1.4)	>.9999
**Number of MM involved, median (IQR)**	**2** (0)	**3** (1)	**2** (0)	**0.0018**
One site involved	8 (9.6)	0 (0)	8 (11.3)	0.5954
Two sites involved	56 (64.5)	5 (41.7)	51 (71.8)	0.0507
**≥ 3 sites involved**	**17 (20.5)**	**6 (50.0)**	**11 (15.5)**	**0.0184**
Blisters (%)				
**Mouth**	**26 (31.3)**	**8 (66.7)**	**18 (25.3)**	**0.0074**
**Skin**	**60 (72.3)**	**5 (41.7)**	**55 (77.5)**	**0.0265**
Genital	3 (3.6)	0 (0)	3 (4.2)	>.9999
LP lesions (%)				
Mouth	61 (73.5)	11 (91.7)	50 (70.4)	0.1681
**Skin**	**62 (74.7)**	**5 (41.7)**	**57 (80.3)**	**0.0127**
**Genital**	**9 (10.8)**	**4 (33.3)**	**5 (7.0)**	**0.0221**
LP or AIBD lesions (%)				
**Mouth**	**49 (60.5)**	**11 (91.7)**	**38 (55.1)**	**0.0230**
**Genital**	**15 (18.5)**	**7 (58.3)**	**8 (11.6)**	**0.0006**
Histology (%)				
AIBD	64 (77.1)	8 (66.7)	56 (78.9)	0.4570
LP	83 (100)	12 (100)	71 (100)	*na*
Circulating auto-antibodies (whatever the method) (%)				
Performed	67 (80.7)	12 (100)	55 (77.5)	0.1106
Positive	54 (80.6)	8 (66.7)	46 (83.7)	0.3421
IFI/IFI-SSS (%)				
**Performed**	**58 (69.9)**	**12** (100)	**46 (64.8)**	**0.0147**
**Positive**	**37 (63.8)**	**3 (25.0)**	**34 (73.9)**	**0.0049**
Blot (%)				
Performed	19 (22.9)	3 (25.0)	16 (22.5)	>.9999
**Full BP180 (n=19)**	**14 (73.7)**	**0** (0)	**14 (87.5)**	**0.0103**
BP180 NC16A (n=10)	6 (60.0)	*nd*	6 (60)	*na*
BP180 Cterm (n=10)	2 (20.0)	*nd*	2 (20)	*na*
BP230 (n=19)	4 (21.1)	0 (0)	4 (25.0)	>.9999
ELISA (%)				
**Performed**	**38 (45.8)**	**12** (100)	**26 (36.6)**	**<0.0001**
**BP180 NC16a (n=38)**	**29 (76.3)**	**6 (50.0)**	**23 (88.5)**	**0.0164**
BP230 (n=27)	6 (22.2)	2 (16.7)	4 (26.7)	0.6618
First line treatment (%)	82 (98.8)	12 (100)	70 (98.6)	>.9999
Corticosteroids only	34 (41.5)	0 (0.0)	34 (48.6)	0.0010
Corticosteroids combined	11 (13.4)	1 (8.3)	10 (14.3)	>.9999
Immunosuppressive (ciclo, MTX, AZA, MMF, sirolimus)	8 (9.8)	0 (0)	8 (11.4)	0.5964
**Immunomodulators (DDS/DOX)**	**17 (20.7)**	**11 (91.7)**	**6 (8.6)**	**<0.0001**
First line treatment efficiency	50 (61.02)	7 (58.3)	43 (61.4)	>.9999
Last line treatment				
**Corticosteroids only**	**31 (37.8)**	**0** (0)	**31 (44.3)**	**0.0027**
Corticosteroids combined with IS	15 (18.3)	1 (8.3)	14 (20.0)	0.4509
**Immunomodulators**	**21 (25.6)**	**10 (83.3)**	**11 (15.7)**	**<0.0001**
Immunosuppressive	14 (17.1)	3 (25.0)	11 (15.7)	0.4216
Rituximab	3 (3.7)	2 (16.7)	1 (1.4)	0.0547
**Retinoids**	**5 (6.1)**	**3 (25.0)**	**2 (2.9)**	**0.0208**
Last line efficiency n=81	68 (84.0)	9 (75.0)	59 (85.5)	0.3975
Status	81	12	69	
CR	48 (59.3)	5 (41.7)	43 (62.3)	0.2130
aCR	5 (6.2)	3 (25.0)	2 (2.89)	0.0215
CD	14 (17.3)	1 (8.3)	13 (18.9)	0.6809
PR	3 (3.7)	0 (0)	3 (4.3)	>.9999
Failure	11 (13.6)	3 (25.0)	8 (11.6)	0.3549
Death reported	5 (6.2)	0 (0)	5 (7.3)	>.9999
Follow-up duration, median in months (IQR)	=15.0 (24.5)	66.0 (60.0)	10.5 (19.5)	<0.0001

MMLPP, mucous membrane lichen planus pemphigoid; IQR, interquartile range; LP, Lichen Planus; AIBD auto-immune blistering disease; NT, nose and throat;MM, mucous membrane; IFI, indirect immunofluorescence; IFI-SSS, IFI on salt split skin; Blot; western-blot; ELISA, Enzyme-Linked Immunosorbent Assay; IS, immunosuppressive; Ciclo, ciclosporine; MTX, methotrexate; AZA, azathioprine; MMF, mycophenolate mofetil; DDS, dapsone; DOX, doxycycline; CR, complete remission; aCR, almost complete remission; CD, controlled disease; PR, partial remission.

Bold values are statistically significant.

Regarding purely cutaneous LPP (**Table 5**), 51 of 61 patients (83.6%) presented histological features of subepidermal AIBD. All DIF results were positive. Circulating autoantibodies were detected in 90.5% of patients, and the sera of 84.4% of patients were positive for IIF on skin and IIF-SSS. ELISA BP180NC16A was positive in 89.5% of patients who underwent the test, and ELISA BP230 was positive in 25% of patients. IB also showed more anti-BP180 autoantibodies (88.9%) than anti-BP230 (11.1%). Most first-line treatments were corticosteroids (54.2% alone and 18.6% in combination with other drugs); immunosuppressive and immunomodulatory drugs were used in 8.5% and 17% of patients, respectively, whereas drug cessation was reported in 93.3% of drug-induced skin LPP. Remission was achieved in 84.7% of cases at the end of follow-up (median duration, 9 months).

**Table 5:** Comparison between the LP-MMP cases (our series + literature) and cutaneous LPP cases from the literature.

Variables	All patients N = 144	Mucous LPP our serie + litterature N = 83	Cutaneous LPP litterature N = 61	p-value
Female gender, N (%)	89 (61.8)	56 (64.5)	33 (54.1)	0.1027
Age at diagnosis (years), median (IQR)	55.0 (33.5)	58.0 (23.0)	47.0 (52.5)	0.0721
Diabetes (%)	17 (11.8)	9 (10.9)	8 (13.1)	0.8760
Chronic viral Hepatitis (%)	4 (2.8)	3 (3.6)	1 (1.6)	0.6375
Cancer (%)	23 (16.0)	15 (18.1)	8 (13.1)	0.5672
Drug induction (%)	24 (16.7)	10 (12.0)	14 (23.0)	0.0828
Order of diagnosis (%)	136 (94.4)	77 (92.8)	59 (96.7)	0.4673
LP first	98 (72.1)	57 (74.0)	41 (69.5)	0.5592
AIBD first	4 (2.9)	4 (5.2)	0 (0)	0.1326
At once	34 (25.0)	16 (20.8)	18 (30.5)	0.1941
Clinical presentation (%)				
**Skin involvement**	**127 (88.2)**	**66 (79.5)**	**61** (100)	**<0.0001**
**Skin blisters (%)**	**118 (81.9)**	**60 (72.3)**	**58 (95.1)**	**0.0003**
**Skin LP lesion (%)**	**122 (84.7)**	**62 (74.7)**	**60 (98.4)**	**<0.0001**
Histology (%)				
AIBD	115 (79.9)	64 (77.1)	51 (83.6)	0.3367
LP	144 (100)	83 (100)	61 (100)	na
Circulating antibodies (%)				
Performed	109 (75.7)	67 (80.7)	42 (68.9)	0.1008
Positive	92 (84.4)	54 (80.6)	38 (90.5)	0.1884
IFI/IFI-SSS (%)				
**Performed**	**90 (62.5)**	**58 (69.9)**	**32 (52.5)**	**0.0329**
Positive	64 (71.1)	37 (63.8)	27 (84.4)	0.0521
Blot Performed (%)	28 (19.4)	19 (22.9)	9 (14.8)	0.3144
Full BP180	22 (78.6)	14 (73.7)	8 (88.9)	0.6296
BP180 NC16A (n=16)	7 (50.0)	6 (60.0)	1 (25.0)	0.5594
BP Cterm (n=16)	4 (25.0)	2 (20.0)	2 (33.3)	0.6044
BP230	5 (17.8)	4 (21.1)	1 (11.1)	>.9999
ELISA Performed (%)	57 (39.6)	38 (45.8)	19 (31.1)	0.0760
BP180	45 (80.7)	29 (76.3)	17 (89.5)	0.3041
BP230	8 (22.9)	6 (22.2)	2 (25.0)	>.9999
First line treatment (%)	141 (97.9)	82 (98.8)	59 (96.7)	0.5741
**Corticosteroids only**	**66 (46.8)**	**34 (41.5)**	**32 (54.2)**	**0.1337**
**CS combined**	**22 (15.6)**	**11 (13.4)**	**11 (18.6)**	**0.3986**
Immunosuppressive (ciclo, MTX, AZA, MMF, sirolimus)	13 (9.2)	8 (9.8)	5 (8.5)	>.9999
Immunomodulateurs (DDS/DOX)	27 (19.1)	17 (20.7)	10 (17.0)	0.5734
First line treatment efficiency (%)	91 (64.1)	50 (61.0)	40 (67.8)	0.4057
Last line treatment	141	82	59	
Corticosteroids only	58 (41.1)	31 (37.8)	27 (45.8)	0.3435
Corticosteroids combined with IS	30 (21.3)	15 (18.3)	15 (25.4)	0.3074
Immunomodulateurs	32 (22.7)	21 (25.6)	11 (18.6)	0.3300
Immunosuppressive	24 (17.0)	14 (17.1)	10 (17.0)	09846
Rituximab	4 (2.8)	3 (3.7)	1 (1.7)	0.6398
Retinoids	6 (4.3)	5 (6.1)	1 (1.7)	0.4008
Last line efficiency (n=140)	123 (87.9)	68 (84.0)	55 (93.2)	0.1199
Status (%)	140	48	44	
**Follow-up duration**	**12.0 (18.7)**	**15.0 (24.5)**	**9.0 (7.7)**	**0.0098**
CR	92 (65.7)	48 (59.3)	44 (74.6)	0.0594
aCR	11 (7.9)	5 (6.2)	6 (10.2)	0.5825
**CR+ aCR**	**103 (73.6)**	**53 (65.4)**	**50 (84.7)**	**0.0180**
CD	18 (12.9)	14 (17.3)	4 (6.8)	0.0776
PR	3 (2.1)	2 (2.4)	1 (1.6)	>.9999
**Failure**	**13 (9.3)**	**11 (13.6)**	**2 (3.4)**	**0.0441**
Death reported (%) n=140	6 (4.3)	5 (6.2)	1 (1.7)	0.4009

LPP, lichen planus pemphigoid; IQR, interquartile range; LP, Lichen Planus; AIBD auto-immune blistering disease; IFI, indirect immunofluorescence; IFI-SSS, IFI on salt split skin; Blot; western-blot; ELISA, Enzyme-Linked Immunosorbent Assay; Ciclo, ciclosporine; MTX, methotrexate; AZA, azathioprine; MMF, mycophenolate mofetil; DDS: dapsone; DOX, doxycycline; CR, complete remission; aCR, almost complete remission; CD, controlled disease; PR, partial remission.

Bold values are statistically significant.

## 5 Discussion

LPP is rare, with an annual incidence in Kuwait (20) estimated at 0.3 per million. Epidemiological studies in France, Germany, Greece, Serbia, and Singapore have not identified any cases of LPP among patients with AIBD, because LPP is rare and not always considered to be a part of the classic list of AIBD (5, 21–25). However, patients with LPP are continuously identified and 132 case reports and small series have been published since 1975; moreover, LPP has been returning to the center stage because of antiPD1/PDL1 induced cases (26–31).

All patients diagnosed with LPP at our center had predominant or exclusive MM involvement. This may have been influenced by the specific recruitment of our referral AIBD center, which specializes in MMP. Therefore, we reported the largest case series of patients with LPP and exclusive or predominant MM involvement.

Our patients did not present any significant differences from those in the literature in terms of age, female predominance, or percentage of comorbidities (particularly cancer) (**Tables 4**, **5**). Approximately half of the LPP cases with a history of malignancy were suspected to be induced by oncological treatment. In the remaining patients, the cancer was in remission, except in one case of LPP associated with multiple keratoacanthomas and colon carcinoma (32). Two patients in the literature review had anti-laminin 332 autoantibodies, which are often associated with cancer (9), but none were detected in the cases reported in this study.

Patients with LPP are typically young, with a median age of 48.9 years at LPP diagnosis, compared with patients with BP (median age, 80 years) (33) and MMP (median age, 60–80 years) (34). Among our cases, one was induced by pembrolizumab (anti-PD1), as observed in nine other recently reported cases of LPP with anti PD1/PDL1 immunotherapies in the literature (26–31).

In clinical presentation, our cases differed significantly from those reported in the literature because they had exclusive or predominant MM lesions (100% vs. 54%; at least two MM involved or MM lesions alone; 100% vs. 31%; **Tables 4**, **5**; **Annex 1**). In comparison with LPP patients with MM involvement in the literature (**Table 4**), we observed more patients with three or more affected sites (50% vs. 15%), more genital involvement (75% vs. 18.3%), and more nasal or pharyngeal (33% vs. 4.2%) involvement. Our patients had less skin involvement (such as blisters and lichenoid lesions). The least frequently affected site in our series was conjunctiva, as also reported in the literature (three patients, 0.04%); however, we did not report any oesophageal involvement, whereas one such case was described in the literature (19, 35, 36) (**Table 4**). Significantly more non-specific lesions were observed in MM in our series compared with the literature, possibly because of the absence of data collection (**Table 4**).

All IEM performed in our study (11 of 12) revealed autoimmune deposits (IgG and/or IgA) on semi-thin sections. Notably, it enabled the detection of deposits not visualized on DIF in four patients, suggesting that IEM might be more sensitive than DIF and of particular interest in diagnosing LPP in patients with negative DIF (37). IEM sensitivity has never been clearly compared with DIF sensitivity, because this technique is usually used to show ultrastructural localization of target antigens and provide an accurate diagnosis of subepithelial AIBD with positive DIF (38). However, two studies on ocular MMP suggested that IEM is more sensitive than DIF (100% sensitivity vs. 67% in a small series of MMP with ocular involvement, or 50% in cases of pure ocular MMP) (37, 39).

In nine of our cases and eight in the literature, ultrastructural deposits were found on the ultrathin sections, in 63% of cases on HD and LL, as in BP, and in 37% of cases on LD and LL, as in MMP (**Table 2**) (19, 40), showing that target antigens are heterogeneous in LPP, in both MMs and skin.

Regarding serological analyses, a significant difference was observed, with more positive skin IIF/IIF-SSS results in the literature compared with our study (73.9% vs. 25%). ELISAs showed the presence of circulating IgG autoantibodies against the BP180-NC16A epitope in six of the 12 (50%) patients in our study, a percentage significantly lower than that observed in the literature [88.5%] (**Table 4**). However, these results do not imply that LPP is the association of LP and BP; as demonstrated by Kromminga et al. (6), LPP sera generally show reactivity with different BP180 fragments, comprising D514–L565, and lack binding to E490–R507 in the NC16A domain, whereas the sera of most BP and PG patients bound to fragments representing amino acids E490–G532 also present in the NC16A domain. Furthermore, IB studies showed IgG autoantibodies to a 200 kDa molecular weight antigen compatible with the laminin 332 alpha 3 chain in one of our patients, as previously reported in two cases in the literature (9, 13). Finally, serological studies were negative in five of our patients. In the literature, ELISA revealed that some patients were negative for BP180–NC16A and/or IB for BP180 and/or BP180–NC16A recombinant protein, but positive in IB for the C-terminal portion, as found in some patients with MMP (41, 42). Unfortunately, we did not have the opportunity to perform IB using C-terminal BP180. Notably, three of the five seronegative patients had immune deposits on the LD of the IEM, consistent with autoantibodies directed against the C-terminal portion of BP180.

The median time between the first symptoms and the diagnosis of LPP in our case series was 5.4 years (range: 0.7–19 years). In most patients, the lichenoid component preceded the first evidence of autoimmune blistering (mean of 4.75 years). The delay between the first lesions and LPP diagnosis might be explained by the presence of mucosal non-specific elementary lesions that may result from LP and/or AIBD (erosions, erythema, atrophy, and synechiae), and LPP was not suspected until typical blisters appeared. Moreover, LPP diagnosis not only requires immunological findings with BMZ autoimmune deposits on DIF or IEM but also histological findings such as lichenoid infiltrates and ideally subepidermal blisters, which sometimes require multiple biopsies at two different sites, especially when AIBD and lichenoid lesions are uncoupled. Therefore, diagnosis can be delayed if histological analyses are not performed on accurate lesions or if DIF or IEM are not systematically performed.

Given the lack of knowledge about the disease; the difficult clinical presentation, especially in case of MM involvement; the often long delay between the appearance of lichenoid and bullous lesions; the difficult diagnosis requiring multiple biopsies; and the low positive rate of complementary blood tests, LPP is probably under-diagnosed. The delay in diagnosis is also a consequence of the lack of awareness of the disease in general practice. In addition, the presence of LP before AIBD lesions, observed in most cases in our series and the literature, corroborates the hypothesis of exposure to normally unexposed BMZ antigens caused by lichenoid lesions. This immunopathological hypothesis regarding the onset of LPP, first proposed by Stingl (2), has been widely discussed in literature (2, 43). The emergence of autoantibodies in LPP appears to be associated with T-cell–mediated lichenoid inflammation, and the LPP phenotype could be the consequence of two kinds of immune responses to BP180, a Th1-response inducing lichenoid lesions and Th2-response inducing autoantibodies and bullous lesions (44). The mechanisms responsible for the relapse and lichenoid or bullous features have not been completely understood. As highlighted in our study, patients with lichenoid or bullous components experienced relapse. This suggests ongoing T- or B-cell–mediated autoimmunity and possibly individual differences in the pathophysiological pathways underpinning relapses. However, as patients received maintenance therapeutics, the differences in the components of relapse could be modulated by the higher efficacy of therapeutics on these pathways. Notably, the highest lichenoid-based relapse rates suggest a stronger therapeutic effect on the blistering component.

The different clinical pictures may be the result of the multiplicity of targeted antigens. Topical corticosteroids alone were not sufficient to control the disease in our series, except in one patient who was only treated with corticosteroid mouthwashes. As our patients shared similarities with patients with MMP because of the predominant mucosal picture, we used immunomodulators (such as dapsone, sulfasalazine, or doxycycline) in the first-line treatment. In non-responding cases, the first used immunomodulatory drug was switched to another or immunosuppressive drugs were added depending on extension/severity, as is usually performed to treat MMP. Among the 11 patients who received systemic drugs, two remained active despite receiving rituximab. Corticosteroids (topical or systemic) were mostly used as first-line treatment in the literature in patients with or without mucosal involvement. Dapsone is rarely prescribed in case reports or series, even in the presence of mucosal lesions. Patients without mucosal lesions were significantly more likely in remission (CR or aCR) than those with mucosal lesions at the end of follow up (84.7% vs. 65.4%), indicating that LPP with MM involvement is more difficult to treat. Mucosal LPP appears to respond well to both corticosteroid and immunomodulatory treatments. However, considering the need for long-term treatment and the well-known side effects of corticosteroids, immunomodulatory molecules appear to be more appropriate (**Table 5**). Most of our patients who received dapsone reached control or remission without systemic corticosteroids or immunosuppressive drugs (67%).

Immunomodulatory drugs could be considered a first-line treatment for active mucosal lesions, such as MMP, and as a steroid-sparing agent for this chronic disease (median follow-up duration of 5.5 years in our series). Two severe patients were treated by rituximab. The B-cell depletion therapy induced by rituximab might be of more interest in LPP considering the reported efficacy in AIBD, such as pemphigus and BP (45). Notably, rituximab was found to deplete circulating B cells and, thus, serum level of pathogenic auto-antibodies in pemphigus (46) by causing a reduction of B-cell and T-cell cross talk implicated in T-cell activation. Accordingly, rituximab was found to decrease circulating autoreactive T cells in pemphigus (47, 48), and long-lasting response to rituximab was found to rely on the decrease of DSG-specific T follicular helper cells participating in sustained depletion of memory auto-reactive B cells and DSG antibody-secreting cells (49). Whereas the pathogenic role of IgG anti-BP180 has been validated in BP (50), their pathogenicity is less demonstrated in MMP, but rituximab efficacy was reported in a large series (51). On the other hand, rituximab efficacy has been uncertain in small series of erosive lichen planus without AIBD, whose immunopathology is more T cell driven (52, 53). This may explain why the two patients who received rituximab still had active disease. It would appear that the indirect effect of rituximab on T cells is not sufficient to treat mediated T-cell lichenoid inflammation in LPP.

Thus, LPP with mucosal involvement can be managed as an MMP, which is supported by the nosology used by some authors. These authors diagnosed patients with clinical, histological, and immunological features similar to MMP because it fulfills the MMP criteria if the disease predominantly affects the MMs (54, 55).

The limitations of our study include its retrospective design and incomplete serological data, particularly the absence of IB using the C-terminal region of BP180.

In conclusion, LPP with mucosal lesions is clinically and immunologically heterogeneous and difficult to diagnose. Diagnosis often requires biopsy, particularly when lichenoid and bullous lesions are observed at different sites. Immunological studies have identified diverse target antigens, such as those in LPP with exclusive skin lesions. Our results suggest that immunomodulators represent an alternative first-line treatment for patients with predominant MM. Larger studies are necessary to clarify the accurate therapeutic strategies depending on disease severity.

## Data availability statement

The original contributions presented in the study are included in the article/**Supplementary Material**. Further inquiries can be directed to the corresponding author.

## Ethics statement

The studies involving humans were approved by Comité local d’éthique de l’hôpital Avicenne (CLEA)-2022-240. The studies were conducted in accordance with the local legislation and institutional requirements. The participants provided their written informed consent to participate in this study. Written informed consent was obtained from the individual(s), and minor(s)’ legal guardian/next of kin, for the publication of any potentially identifiable images or data included in this article.

## Author contributions

LC, CL-V, and CP-S contributed to the conception and the design of the study. LC, CL-V, GB, MA, FC, and CP-S handled the patients’ daily care and dermatological data collection. IS was involved in ENT assessment and data collection. SD performed ophthalmological assessment and data collection. FP and I-YS performed stomatological assessment and data collection. FM and SG-M performed the serum immunological analyses. AM and BA performed histopathological and direct immunofluorescence analyses. CP-S performed direct immunoelectron microscopy. LC, GB, CL-V, and CP-S reviewed the charts of the patients and organized the databases. GB performed the statistical analysis. LC wrote the first draft of the manuscript and figures. All authors contributed to the article and approved the submitted version.

## References

[1] Kaposi M. Lichen ruber pemphigoides. Arch J Dermatol Syph. (1892) 24:343–6.

[2] Stingl G Hobular K. Coexistence of lichen planus and bullous pemphigoid. A immunopathological study. Br J Dermatol. (1975) 93:313–20. doi: 10.1111/j.1365-2133.1975.tb06497.x 1103935

[3] Cognat T Gayrard L Adam C Balmex B MaChado P Nicolas JF. Lichen plan pemphigoïde [Lichen planus pemphigoides]. Ann Dermatol Venereol. (1991) 118:387–90. 1897822

[4] Ogg GS Bhogal BS Hashimoto T Coleman R Barker JN. Ramipril-associated lichen planus pemphigoides. Br J Dermatol. (1997) 136:412–4. 9115928

[5] Zillikens D Caux F Mascaro JM Wesselmann U Schmidt E Prost C. Autoantibodies in lichen planus pemphigoides react with a novel epitope within the C-terminal NC16A domain of BP180. J Invest Dermatol. (1999) 113:117–21. doi: 10.1046/j.1523-1747.1999.00618.x 10417629

[6] Kromminga A Sitaru C Meyer J Arndt R Schmidt E Christophers E. Cicatricial pemphigoid differs from bullous pemphigoid and pemphigoid gestationis regarding the fine specificity of autoantibodies to the BP180 NC16A domain. J Dermatol Sci. (2002) 28:68–75. doi: 10.1016/s0923-1811(01)00144-x 11916132

[7] Inoue Y Adachi A Ueno M Fukumoto T Nishitani N Fujiwara N. Atypical subacute cutaneous lupus erythematosus presenting as lichen planus pemphigoides with autoantibodies to C-terminus of BP180, desmoglein 1 and SS-A/Ro antigen. J Dermatol. (2012) 39:960–2. doi: 10.1111/j.1346-8138.2012.01536.x 22413755

[8] Sekiya A Kodera M Yamaoka T Iwata Y Usuda T Ohzono A. A case of lichen planus pemphigoides with autoantibodies to the NC16a and C-terminal domains of BP180 and to desmoglein-1. Br J Dermatol. (2014) 171:1230–5. doi: 10.1111/bjd.13097 24813536

[9] Fukuda A Himejima A Tsuruta D Koga H Ohyama B Morita S. Four cases of mucous membrane pemphigoid with clinical features of oral lichen planus. Int J Dermatol. (2016) 55:657–65. doi: 10.1111/ijd.12884 26341508

[10] Yoshida S Shiraishi K Yatsuzuka K Mori H Koga H Ishii N. Lichen planus pemphigoides with antibodies against the BP180 C-terminal domain induced by pembrolizumab in a melanoma patient. J Dermatol. (2021) 48:e449–51. doi: 10.1111/1346-8138.16006 34089265

[11] Shimada H Shono T Sakai T Ishikawa K Takeo N Hatano Y. Lichen planus pemphigoides concomitant with rectal adenocarcinoma: fortuitous or a true association? Eur J Dermatol. (2015) 25:501–3. doi: 10.1684/ejd.2015.2619 26394647

[12] Paige DG Bhogal BS Black MM Harper JI. Lichen planus pemphigoides in a child—immunopathological findings. Clin Exp Dermatol. (1993) 18:552–4. doi: 10.1111/j.1365-2230.1993.tb01029.x 8252796

[13] Yoon KH Kim SC Kang DS Lee IJ. Lichen planus pemphigoides with circulating autoantibodies against 200 and 180 kDa epidermal antigens. Eur J Dermatol. (2000) 10:212–4. 10725820

[14] Zaraa I Mahfoudh A Kallel Sellami M Chelly I El Euch D Zitouna M. Lichen planus pemphigoides: four new cases of the literature. Int J Dermatol. (2013) 52:406–12. doi: 10.1111/j.1365-4632.2012.05693.x 23331194

[15] Grootenboer-Mignot S Descamps V Picard-Dahan C Nicaise-Roland P Prost-Squarcioni C Leroux-Villet C. Place of human amniotic membrane immunoblotting in the diagnosis of autoimmune bullous dermatoses. Br J Dermatol. (2010) 162:743–50. doi: 10.1111/j.1365-2133.2009.09566.x 19886889

[16] Murrell DF Marinovic B Caux F Prost C Ahmed R Wozniak K. Definitions and outcome measures for mucous membrane pemphigoid: recommendations of an international panel of experts. J Am Acad Dermatol. (2015) 72:168–74. doi: 10.1016/j.jaad.2014.08.024 25443626

[17] Page MJ McKenzie JE Bossuyt PM Boutron I Hoffmann TC Mulrow CD. Updating guidance for reporting systematic reviews: development of the PRISMA 2020 statement. J Clin Epidemiol. (2021) 134:103–12. doi: 10.1016/j.jclinepi.2021.02.003 33577987

[18] Tauber J Jabbur N Foster CS. Improved detection of disease progression in ocular cicatricial pemphigoid. Cornea. (1992) 11:446–51. doi: 10.1097/00003226-199209000-00015 1424674

[19] Bouloc A Vignon-Pennamen MD Caux F Teillac D Wechsler J Heller M. Lichen planus pemphigoides is a heterogeneous disease: a report of five cases studied by immunoelectron microscopy. Br J Dermatol. (1998) 138:972–80. doi: 10.1046/j.1365-2133.1998.02262.x 9747357

[20] Nanda A Dvorak R Al-Saeed K Al-Sabah H Alsaleh QA. Spectrum of autoimmune bullous diseases in Kuwait. Int J Dermatol. (2004) 43:876–81. doi: 10.1111/j.1365-4632.2004.02292.x 15569006

[21] Bernard P Vaillant L Labeille B Bedane C Arbeille B Denoeux JP. Incidence and distribution of subepidermal autoimmune bullous skin diseases in three French regions. Bullous diseases French study group. Arch Dermatol. (1995) 131:48–52. 7826096

[22] Bertram F Broücker E-B Zillikens D Schmidt E. Prospective analysis of the incidence of autoimmune bullous disorders in Lower Franconia, Germany. J Dtsch Dermatol Ges. (2009) 7:434–40. doi: 10.1111/j.1610-0387.2008.06976.x 19170813

[23] Patsatsi A Lamprou F Kokolios M Stylianidou D Trigoni A Kalampalikis D. Spectrum of autoimmune bullous diseases in Northern Greece. A 4-year retrospective study and review of the literature. Acta Dermatovenerol Croat. (2017) 25:195–201. 29252171

[24] Milinković MV Janković S Medenica L Nikolić M Reljić V Popadić S. Incidence of autoimmune bullous diseases in Serbia: a 20-year retrospective study. J Dtsch Dermatol Ges. (2016) 14:995–1005. doi: 10.1111/ddg.13081 27767273

[25] Wong SN Chua SH. Spectrum of subepidermal immunobullous disorders seen at the National Skin Centre, Singapore: a 2-year review. Br J Dermatol. (2002) 147:476–80. doi: 10.1046/j.1365-2133.2002.04919.x 12207586

[26] Schmidgen MI Butsch F SChadmand-Fischer S Steinbrink K Grabbe S Weidenthaler-Barth B. Pembrolizumab-induced lichen planus pemphigoides in a patient with metastatic melanoma. J Dtsch Dermatol Ges. (2017) 15:742–5. doi: 10.1111/ddg.13272 28622432

[27] Kerkemeyer KLS Lai FYX Mar A. Lichen planus pemphigoides during therapy with tislelizumab and sitravatinib in a patient with metastatic lung cancer. Australas J Dermatol. (2020) 61:180–2. doi: 10.1111/ajd.13214 31808542

[28] Manko S Côté B Provost N. A case of durvalumab-induced lichenoid eruption evolving to bullous eruption after phototherapy: a case report. SAGE Open Med Case Rep. (2021) 9. doi: 10.1177/2050313X21993279 PMC7940716 33747513

[29] Okada H Kamiya K Murata S Sugihara T Sato A Maekawa T. Case of a lichen planus pemphigoides after pembrolizumab therapy for advanced urothelial carcinoma. J Dermatol. (2020) 47:e321–2. doi: 10.1111/1346-8138.15461 32515025

[30] Senoo H Kawakami Y Yokoyama E Yamasaki O Morizane S. Atezolizumab-induced lichen planus pemphigoides in a patient with metastatic non-small-cell lung cancer. J Dermatol. (2020) 47:e121–2. doi: 10.1111/1346-8138.15248 31984550

[31] Boyle MM Ashi S Puiu T Reimer D Sokumbi O Soltani K. Lichen planus pemphigoides associated with PD-1 and PD-L1 inhibitors: a case series and review of the literature. Am J Dermatopathol. (2022) 44:360–7. doi: 10.1097/DAD.0000000000002139 35120032

[32] Hamada T Fujimoto W Okazaki F Asagoe K Arata J Iwatsuki K. Lichen planus pemphigoides and multiple keratoacanthomas associated with colon adenocarcinoma. Br J Dermatol. (2004) 151:252–4. doi: 10.1111/j.1365-2133.2004.06074.x 15270914

[33] Joly P Baricault S Sparsa A Bernard P Bédane C Duvert-Lehembre S. Incidence and mortality of bullous pemphigoid in France. J Invest Dermatol. (2012) 132:1998–2004. doi: 10.1038/jid.2012.35 22418872

[34] Du G Patzelt S van Beek N Schmidt E. Mucous membrane pemphigoid. Autoimmun Rev. (2022) 21:103036. doi: 10.1016/j.autrev.2022.103036 34995762

[35] Murphy GM Cronin E. Lichen planus pemphigoides. Clin Exp Dermatol. (1989) 14:322–4. doi: 10.1111/j.1365-2230.1989.tb01994.x 2686875

[36] Mignogna MD Fortuna G Leuci S Stasio L Mezza E Ruoppa E. Lichen planus pemphigoides, a possible example of epitope spreading. Oral Surg Oral Med Oral Pathol Oral Radiol Endod. (2010) 109:837–43. doi: 10.1016/j.tripleo.2009.12.044 20382044

[37] Demers PE Robin H Prost C Toutblanc M Hoang-Xuan T. Immunohistopathologic testing in patients suspected of ocular cicatricial pemphigoid. Curr Eye Res. (1998) 17:823–7. 9723998

[38] Chan WMM Lee JS Thiam Theng CS Chua SH Boon Oon HH. Narrowband UVB-induced lichen planus pemphigoide. Dermatol Rep. (2011) 3:43. doi: 10.4081/dr.2011.e43 PMC4211512 25386295

[39] Hoang-Xuan T Robin H Demers PE Heller M Toutblanc M Dubertret L. Pure ocular cicatricial pemphigoid. A distinct immunopathologic subset of cicatricial pemphigoid. Ophthalmology. (1999) 106:355–61. doi: 10.1016/S0161-6420(99)90076-3 9951490

[40] Swale VJ Black MM Bhogal BS. Lichen planus pemphigoides: two case reports. Clin Exp Dermatol. (1998) 23:132–5. doi: 10.1046/j.1365-2230.1998.00337.x 9861745

[41] Murakami H Nishioka S Setterfield J Bhogal BS Black MM Zillikens D. Analysis of antigens targeted by circulating IgG and IgA autoantibodies in 50 patients with cicatricial pemphigoid. J Dermatol Sci. (1998) 17:39–44. doi: 10.1016/s0923-1811(97)00067-4 9651827

[42] Nakatani C Muramatsu T Shirai T. Immunoreactivity of bullous pemphigoid (BP) autoantibodies against the NC16A and C-terminal domains of the 180 kDa BP antigen (BP180): immunoblot analysis and enzyme-linked immunosorbent assay using BP180 recombinant proteins. Br J Dermatol. (1998) 139:365–70. doi: 10.1046/j.1365-2133.1998.02396.x 9767278

[43] Hübner F Langan EA Recke A. Lichen planus pemphigoides: from lichenoid inflammation to autoantibody-mediated blistering. Front Immunol. (2019) 10:1389. doi: 10.3389/fimmu.2019.01389 31312198 PMC6614382

[44] Schmidt T Solimani F Pollmann R Stein R Schmidt A Stulberg I. T_H_1/T_H_17 cell recognition of desmoglein 3 and bullous pemphigoid antigen 180 in patients with lichen planus. J Allergy Clin Immunol. (2018) 142:669–72. doi: 10.1016/j.jaci.2018.02.044 29626572

[45] Fang H Li Q Wang G. The role of T cells in pemphigus vulgaris and bullous pemphigoid. Autoimmun Rev. (2020) 19:102661. doi: 10.1016/j.autrev.2020.102661 32942041

[46] Bohelay G Caux F Musette P. Clinical and biological activity of rituximab in the treatment of pemphigus. Immunotherapy. (2021) 13:35–53. doi: 10.2217/imt-2020-0189 33045883

[47] Eming R Nagel A Wolff-Franke S Podstawa E Debus D Hertl M. Rituximab exerts a dual effect in pemphigus vulgaris. J Invest Dermatol. (2008) 128:2850–8. doi: 10.1038/jid.2008.172 18563178

[48] Leshem YA David M Hodak E Waitman DA Vardy D Israeli M. A prospective study on clinical response and cell-mediated immunity of pemphigus patients treated with rituximab. Arch Dermatol Res. (2014) 306:67–74. doi: 10.1007/s00403-013-1355-4 23591742

[49] Maho-Vaillant M Perals C Golinski ML Hébert V Caillot F Mignard C. Rituximab and corticosteroid effect on desmoglein-specific B cells and desmoglein-specific T follicular helper cells in pemphigus. J Invest Dermatol. (2021) 141:2132–40. doi: 10.1016/j.jid.2021.01.031 33766510

[50] Genovese G Di Zenzo G Cozzani E Berti E Cugno M Marzano AV. New insights into the pathogenesis of bullous pemphigoid: 2019 update. Front Immunol. (2019) 10:1506. doi: 10.3389/fimmu.2019.01506 31312206 PMC6614376

[51] Bohelay G Alexandre M Le Roux-Villet C Sitbon I Doan S Soued I. Rituximab therapy for mucous membrane pemphigoid: a retrospective monocentric study with long-term follow-up in 109 patients. Front Immunol. (2022) 13:915205. doi: 10.3389/fimmu.2022.915205 35844526 PMC9281543

[52] Tétu P Monfort JB Barbaud A Francès C Chasset F. Failure of rituximab in refractory erosive lichen planus. Br J Dermatol. (2018) 179:980–1. doi: 10.1111/bjd.16704 29704877

[53] Lagerstedt M Kotaniemi-Talonen L Antonen J Vaalasti A. Erosive vulvo-vaginal lichen planus treated with rituximab: A case report. Int J Gynaecol Obstet. (2022) 156:172–3. doi: 10.1002/ijgo.13814 34214191

[54] Benzaquen M Suter VGA Gschwend M Feldmeyer L Borradori L. Mucous membrane pemphigoid of the oral lichen type: a retrospective analysis of 16 cases. J Eur Acad Dermatol Venereol. (2019) 33:205–7. doi: 10.1111/jdv.15473 30720898

[55] Rashid H Lamberts A Borradori L Alberti-Violetti S Barry RJ Caproni M. European guidelines (S3) on diagnosis and management of mucous membrane pemphigoid, initiated by the European Academy of Dermatology and Venereology—Part I. J Eur Acad Dermatol Venereol. (2021) 35:1750–64. doi: 10.1111/jdv.17397 PMC8457055 34245180

