# Supplementary material for: Lichen planus pemphigoides with predominant mucous membrane involvement: a series of 12 patients and a literature review
Source: Front Immunol. 2024 Apr 15;15:1243566. doi: 10.3389/fimmu.2024.1243566 (PMC11057232; doi:10.3389/fimmu.2024.1243566)
Supplement: Supplementary file 1 [file DataSheet_1.docx]

# Annex 1: Literature Review References

# Sobel S, Miller R, Shatin H. Lichen planus pemphigoides: immunofluorescence findings. Arch Dermatol. (1976) 112:1280–3.

# Saurat JH, Guinepain MT, Didierjean L, Sohier J, Puissant A. Coexistence of lichen planus and bullous pemphigoid: an immunofluorescence study of a "lichen pemphigoide”. Ann Dermatol Venereol. (1977) 104:368–74.

# Hintner H, Tappeiner G, Hönigsmann H, Wolff K. Lichen planus and bullous pemphigoid. Acta Derm Venereol Suppl. (1979) 59:71–6.

# Morel P, Perron J, Crickx B, Barrandon Y, Civatte J. Lichen plan avec dépôts linéaires d'IgG et de C3 à la jonction dermo-épidermique. Dermatologica (1981) 163:117–24.

# Collins NJ, Dowling JP. Lichen planus and bullous pemphigoid. Australas J Dermatol. (1982) 23:9–13.

# Lang PG, Maize JC. Coexisting lichen planus and bullous pemphigoide or lichen planus pemphigoides? J Am Acad Dermatol. (1983) 9:133–40.

# Godard W, Ingargiola F, Tavernier B, Justrabo E, Lambert D. Lichen plan pemphigoïde et PUVA thérapie. Ann Dermatol Venerol. (1983) 110:69–74.

# Miyagawa S, Ohi H, Muramatsu T, Okuchi T, Shirai T, Sakamoto K. Lichen planus pemphigoides-like lesions induced by cinnarizine. Br J Dermatol. (1985) 112:607–13.

# Prost C, Tesserand F, Laroche L, Dallot A, Verola O, Morel P, et al. Lichen planus pemphigoides: an immuno-electron microscopic study. Br J Dermatol. (1985) 113:31–6.

# Flageul B, Foldes S, Wallach D, Vignon-Pennamen MD, Cottenot F. Captopril-induced lichen planus pemphigoides with pemphigus-like features. A case report. Dermatologica (1986) 173:248–55.

# Oomen C, Temmerman L, Kunt A. Lichen Planus Pemphigoides. Clin Exp Dermatol. (1986) 11:92–6.

# Allen CM, Camisa C, Grimwood R. Lichen planus pemphigoides: report of a case with oral lesions. Oral Surg Oral Med Oral Pathol. (1987) 63:184–8.

# Gawkrodger DJ, Stavropoulos PG, McLaren KM, Buxton PK. Bullous lichen planus and lichen planus pemphigoides—clinico-pathological comparisons. Clin Exp Dermatol. (1989) 14:150–3.

# Lotem M, Ingber A, Sandbank M, Hazaz B. Lichen planus pemphigoides with features of lichen planus and pemphigus vulgaris. Arch Dermatol. (1989) 125:707–8.

# Murphy GM, Cronin E. Lichen planus pemphigoides. Clin Exp Dermatol. (1989) 14:322–4.

# Okochi H, Nashiro K, Tsuchida T, Seki Y, Tamaki K. Lichen planus pemphigoides: case report and results of immunofluorescence and immunoelectron microscopic study. J Am Acad Dermatol. (1990) 22:626–31.

# Archer CB, Cronin E, Smith NP. Diagnosis of lichen planus pemphigoides in the absence of bullae on normal-appearing skin Clin Exp Dermatol. (1992) 17:433–6.

# Cognat T, Gayrard L, Adam C, Balmex B, Machado P, Nicolas JF, Moulin G. Lichen plan pemphigoïde [Lichen planus pemphigoides]. Ann Dermatol Venerol. (1991) 118:387–90.

# Davis AL, Bhogal BS, Whitehead P, Frith P, Murdoch ME, Leigh IM, et al. Lichen planus pemphigoides: its relationship to bullous pemphigoid. Br J Dermatol. (1991) 125:263–71.

# Borrego Hernando L, Vanaclocha Sebastián F, Hergueta Sánchez J, Ortiz Romero P, Iglesias Diez L. Lichen planus pemphigoides in a 10-year-old girl. J Am Acad Dermatol. (1992) 26:124–5.

# Maceyko RF, Camisa C, Bergfeld WF, Valenzuela R. Oral and cutaneous lichen planus pemphigoides. J Am Acad Dermatol. (1992) 27:889–92.

# Paige DG, Bhogal BS, Black MM, Harper JI. Lichen planus pemphigoides in a child—immunopathological findings. Clin Exp Dermatol. (1993) 18:552–4.

# Joshi RK, Atukorala DN, Abanmi A, al Awadi T. Lichen planus pemphigoides. Is it a separate entity? Br J Dermatol. (1994) 130:537–8.

# Fivenson DP, Kimbrough TL. Lichen planus pemphigoides: combination therapy with tetracycline and nicotinamide. J Am Acad Dermatol. (1997) 36:638–40.

# Ogg GS, Bhogal BS, Hashimoto T, Coleman R, Barker JN. Ramipril-associated lichen planus pemphigoides. Br J Dermatol. (1997) 136:412–4.

# Reynaers A, Degreef H. Severe erosive stomatitis: association with immunological diseases? Dermatology (1997) 194:411–5.

# Bouloc A, Vignon-Pennamen MD, Caux F, Teillac D, Wechsler J, Heller M, et al. Lichen planus pemphigoides is a heterogeneous disease: a report of five cases studied by immunoelectron microscopy. Br J Dermatol. (1998) 138:972–80.

# Sapadin AN, Phelps RG, Fellner MJ, Kantor I. Lichen planus pemphigoides presenting with a strikingly unilateral distribution. Int J Dermatol. (1998) 37:942–6.

# Flageul B, Hassan F, Pinquier L, Blanchet-Bardon C, Dubertret L. Lichen pemphigoïde associé à une hépatite B évolutive chez un enfant. [Lichen pemphigoid associated with developing hepatitis B in a child]. Ann Dermatol Venereol. (1999) 126:604–7.

# Skaria M, Salomon D, Jaunin F, Friedli A, Saurat JH, Borradori L. IgG autoantibodies from a lichen planus pemphigoides patient recognize the NC16A domain of the bullous pemphigoid antigen 180. Dermatology (1999) 199:253–5.

# Swale VJ, Black MM, Bhogal BS. Lichen planus pemphigoides: two case reports. Clin Exp Dermatol. (1998) 23:132–5.

# Hsu S, Ghohestani RF, Uitto J. Lichen planus pemphigoides with IgG autoantibodies to the 180 kd bullous pemphigoid antigen (type XVII collagen). J Am Acad Dermatol. (2000) 42:136–41.

# Yoon KH, Kim SC, Kang DS, Lee IJ. Lichen planus pemphigoides with circulating autoantibodies against 200 and 180 kDa epidermal antigens. Eur J Dermatol. (2000) 10:212–4.

# Dermiçay Z, Baykal C, Demirkesen C. Lichen planus pemphigoides: report of two cases. Int J Dermatol. (2001) 40:757–9.

# Sakuma-Oyama Y, Powell AM, Albert S, Oyama N, Bhogal BS, Black MM. Lichen planus pemphigoides evolving into pemphigoid nodularis. Clin Exp Dermatol. (2003) 28:613–6.

# Stoebner PE, Michot C, Ligeron C, Durand L, Meynadier J, Meunier L. Lichen plan pemphigoïde induit par la simvastatine [Simvastatin-induced lichen planus pemphigoides]. Ann Dermatol Venereol. (2003) 130:187–90.

# Hamada T, Fujimoto W, Okazaki F, Asagoe K, Arata J, Iwatsuki K. Lichen planus pemphigoides and multiple keratoacanthomas associated with colon adenocarcinoma. Br J Dermatol. (2004) 151:252–4.

# Harting MS, Hsu S. Lichen planus pemphigoides: a case report and review of the literature. Dermatol Online J. (2006) 12:10.

# Zhu YI, Fitzpatrick JE, Kornfeld BW. Lichen planus pemphigoides associated with ramipril. Int J Dermatol. (2006) 45:1453–5.

# Maoz KB, Brenner S. Lichen planus pemphigoides triggered by narrowband UVB, paracetamol and ibuprofen with autoantibodies to 130kDa antigen. Skinmed (2008) 7:33–6.

# Ben Salem C, Chenguel L, Ghariani N, Denguezli M, Hmouda H, Bouraoui K. Captopril-induced lichen planus pemphigoides. Pharmacoepidemiol Drug Saf. (2008) 17:722–4.

# Cohen DM, Ben-Amitai D, Feinmesser M, Zvulunov A. Childhood lichen planus pemhigoides: a case report and review of the literature. Pediatr Dermatol. (2009) 26:569–74.

# Jensen AØ, Steiniche T, Veien NK, Deleuran MS. Lichen planus pemphigoides in a 6-year-old child. Acta Paediatr. (2009) 98:2–3.

# Skvara H, Stingl G. Lichenoid eruption with single plantar blisters: a very rare case of lichen planus pemphigoides. J Eur Acad Dermatol Venereol. (2009) 23:596–7.

# Xu HH, Xiao T, He CD, Jin GY, Wang YK, Gao XH, et al. Lichen planus pemphigoides associated with Chinese herbs. Clin Exp Dermatol. (2009) 34:329–32.

# Mignogna MD, Fortuna G, Leuci S, Stasio L, Mezza E, Ruoppo E. Lichen planus pemphigoides, a possible example of epitope spreading. Oral Surg Oral Med Oral Pathol Oral Radiol Endod. (2010) 109:837–43.

# Barnadas MA, Roé E, Dalmau J, Alomar A, Martínez L, Gelpí C. Lichen planus pemphigoides: detection of anti-BP 180 antibodies by ELISA and immunoblotting tests. J Eur Acad Dermatol Venereol. (2010) 24:1360–1.

# Anand D, Bernardin R, Rubin AI. Blisters and plaques on the extremities. What is your diagnosis? Lichen planus pemphigoides Int J Dermatol. (2011) 50:147–9.

# İlknur T, Akarsu S, Uzun S, Özer E, Fetil E. Heterogeneous disease: a child case of lichen planus pemphigoides triggered by varicella. J Dermatol. (2011) 38:707–10.

# Chan WMM, Lee JS, Thiam Theng CS, Chua SH, Boon Oon HH. Narrowband UVB-induced lichen planus pemphigoide. Dermatol Rep. (2011) 3:43. doi:10.4081/dr.2011.e43

# Duong B, Marks S, Sami N, Theos A. Lichen planus pemphigoides in a 2-year-old girl: response to treatment with methotrexate. J Am Acad Dermatol. (2012) 67:154–6. Doi: 10.1016/j.jaad.2011.12.024

# Rosmaninho A, Sanches M, Oliveira A, Alves R, Selores M. Lichen planus pemphigoides induced by a weight reduction drug. Cutan Ocul Toxicol. (2011) 30:306–8.

# Conde Fernandes I, Pinto Almeida T, Mendes I, Cunha Velho G, Alves R, Selores M. Lichen planus pemphigoides in a child. Eur J Dermatol. (2012) 22:570–1.

# Inoue Y, Adachi A, Ueno M, Fukumoto T, Nishitani N, Fujiwara N, et al. Atypical subacute cutaneous lupus erythematosus presenting as lichen planus pemphigoides witch autoantibodies to C-terminus of BP180, desmoglein 1 and SS-A/Ro antigen. J Dermatol. (2012) 39:960–2.

# Laureano A, Rafael M, Marques Pinto G, Cardoso J. Lichen planus pemphigoides possibly induced by hormone therapy. Eur J Dermatol. (2013) 23:903–4.

# Washio K, Nakamura A, Fukuda S, Hashimoto T, Horikawa T. A case of lichen planus pemphigoides successfully treated with a combination of cyclosporine A and prednisolone. Case Rep Dermatol. (2013) 5:84–7.

# Zaraa I, Mahfoudh A, Sellami MK, Chelly I, El Euch D, Zitouna M, et al. Lichen planus pemphigoides: four new cases and a review of the literature. Int J Dermatol. (2013) 52:406–12.

# Goldscheider I, Herzinger T, Varga R, Eming R, Ruzicka T, Flaig MJ, et al. Childhood lichen planus pemphigoides: report of two cases treated successfully with systemic glucocorticoids and dapsone. Pediatr Dermatol. (2014) 31:751–3.

# Mohanarao TS, Kumar GA, Chennamsetty K, Priyadarshini T. Childhood lichen planus pemphigoides triggered by chickenpox. Indian Dermatol Online J. (2014) 5:S98–100.

# Sekiya A, Kodera M, Yamaoka T, Iwata Y, Usuda T, Ohzono A, et al. A case of lichen planus pemphigoides with autoantibodies to the NC16a and C-terminal domains of BP180 and to desmoglein-1. Br J Dermatol. (2014) 171:1230–5.

# Jang SH, Yun SJ, Lee SC, Lee JB. Lichen planus pemphigoides associated with chronic hepatitis B virus infection. Clin Exp Dermatol. (2015) 40:868–71.

# Kumar P, Savant SS, Das A, Hassan S, Barman PD. Lichen planus pemphigoides presenting preferentially over preexisting scars: a rare instance of isotopic phenomenon. Indian J Dermatol. (2015) 60:596–9.

# Shimada H, Shono T, Sakai T, Ishikawa K, Takeo N, Hatano Y, et al. Lichen planus pemphigoides concomitant with rectal adenocarcinoma: fortuitous or a true association? Eur J Dermatol. (2015) 25:501–3.

# Sultan A, Stojanov IJ, Lerman MA, Kabani S, Haber J, Freedman J, et al. Oral lichen planus pemphigoides: a series of four cases. Oral Surg Oral Med Oral Pathol Oral Radiol. (2015) 120:58–68.

# Fukuda A, Himejima A, Tsuruta D, Koga H, Ohyama B, Morita S, et al. Four cases of mucous membrane pemphigoid with clinical features of oral lichen planus. Int J Dermatol. (2016) 55:657–65.

# Malakar S, Saha A. Successful treatment of resistant lichen planus pemphigoides with cyclosporine: a new hope. Indian J Dermatol. (2016) 61:112–4.

# Rullán J, Diaz NC, Vazquez-Botet M. Lichen planus pemphigoides associated with pregnancy mimicking pemphigoid gestationis. Cutis (2016) 97:20–4.

# Tan AJ, Vaidya S. Non-bullous lichen planus pemphigoides possibly induced by venlafaxine. Australas J Dermatol. (2016) 57:154–5.

# Fujii M, Takahashi I, Honma M, Ishida-Yamamoto. A. Bullous lichen planus accompanied by elevation of serum anti-BP180 autoantibody: a possible transitional mechanism to lichen planus pemphigoides. J Dermatol. (2017) 44:e124–e125. doi: 10.1111/1346-8138.13732

# Knisley RR, Petropolis AA, Mackey VT. Lichen planus pemphigoides treated with ustekinumab. Cutis (2017) 100:415–8.

# Loyal J, Rashtak S. Vulvar lichen planus pemphigoides. Int J Womens Dermatol. (2017) 3:225–7.

# Onprasert W, Chanprapaph K. Lichen planus pemphigoides induced by enalapril: a case report and a review of literature. Case Rep Dermatol. (2017) 9:217–24.

# Schmidgen MI, Butsch F, Schadmand-Fischer S, Steinbrink K, Grabbe S, Weidenthaler-Barth B, et al. Pembrolizumab-induced lichen planus pemphigoides in a patient with metastatic melanoma. J Dtsch Dermatol Ges. (2017) 15:742–5.

# Matos-Pires E, Campos S, Lencastre A, João A, Mendes-Bastos P. Lichen planus pemphigoides. J Dtsch Dermatol Ges. (2018) 16:335–7.

# Mizawa M, Makino T, Hayashi M, Furukawa F, Shimizu T. A case of lichen planus pemphigoides with palmoplantar keratoderma. Eur J Dermatol. (2018) 28:100–1.

# Ondhia C, Kaur C, Mee J, Natkunarajah J, Singh M. Lichen planus pemphigoides mimicking toxic epidermal necrolysis. Am J Dermatopathol. (2019) 41:144–7. doi: 10.1097/DAD.0000000000001461

# Orozco-Anahuati AP, Morgado-Carrasco D, Fustà-Novell X, Mascaró JM Jr. Lichen planus pemphigoides with complete clinical response after ciclosporin therapy. Australas J Dermatol. (2019) 60:254–6. doi: 10.1111/ajd.13013

# Sato Y, Fujimura T, Mizuashi M, Aiba S. Lichen planus pemphigoides developing from patient with non-small-cell lung cancer treated with nivolumab. J Dermatol. (2019) 46:e374–e375. doi: 10.1111/1346-8138.14906

# Stoopler ET, Charmelo-Silva S, Bindakhil M, Alawi F, Sollecito TP. Oral lichen planus pemphigoides: three cases of a rare entity. Am J Dermatopathol. (2020) 42:467–9.

# Strickley JD, Vence LM, Burton SK, Callen JP. Nivolumab-induced lichen planus pemphigoides. Cutis (2019) 103:224–6.

# Bosch-Amate X, Riquelme-McLoughlin C, Morgado-Carrasco D, Rojano-Fritz L, Iranzo-Fernandez P. Report of two cases of mucous membrane pemphigoid with frontal fibrosing alopecia: a variant of lichen planus pemphigoides or an incidental finding? Clin Exp Dermatol. (2020) 45:727–31.

# Brennan M, Baldissano M, King L, Gaspari AA. Successful use of rituximab and intravenous gamma globulin to treat checkpoint inhibitor-induced severe lichen planus pemphigoides. Skinmed (2020) 18:246–9.

# Kerkemeyer KLS, Lai FYX, Mar A. Lichen planus pemphigoides during therapy with tislelizumab and sitravatinib in a patient with metastatic lung cancer. Australas J Dermatol. (2020) 61:180–2.

# Kerkemeyer KL, Pinczewski J, Sinclair R. Successful treatment of recalcitrant lichen planus pemphigoides with tildrakizumab. Australas J Dermatol. (2020) 61:e366–e368. doi: 10.1111/ajd.13263

# Kwon CW, Murthy RK, Kudchadkar R, Stoff BK. Pembrolizumab-induced lichen planus pemphigoides in a patient with metastatic Merkel cell carcinoma. JAAD Case Rep. (2020) 6:1045–7.

# Lamberts A, Diercks GFH, Pas HH, Horváth B. Non-bullous lichen planus pemphigoides: a case report. Acta Derm Venereol. (2020) 100:adv00156. doi: 10.2340/00015555-3523

# Mangin MA, Kanitakis J, Jullien D, Lesort C. Dermpath & Clinic: Lichen planus pemphigoides. Eur J Dermatol. (2020) 30:211–3.

# Okada H, Kamiya K, Murata S, Sugihara T, Sato A, Maekawa T, et al. Case of a lichen planus pemphigoides after pembrolizumab therapy for advanced urothelial carcinoma. J Dermatol. (2020) 47:e321–e322. doi: 10.1111/1346-8138.15461

# Pizzatti L, Ferreli C, Conti B, Atzori L, Pinna G, Pilloni L, et al. Childhood erythrodermic lichen planus pemphigoides after nonavalent human papillomavirus vaccination. JAAD Case Rep. (2020) 6:431–3.

# Senoo H, Kawakami Y, Yokoyama E, Yamasaki O, Morizane S. Atezolizumab-induced lichen planus pemphigoides in a patient with metastatic non-small-cell lung cancer. J Dermatol. (2020) 47:e121–e122 doi: 10.1111/1346-8138.15248

# Manko S, Côté B, Provost N. A case of durvalumab-induced lichenoid eruption evolving to bullous eruption after phototherapy: a case report. SAGE Open Med Case Rep. (2021) 9. doi: 10.1177/2050313X21993279

# Rajaintharan S, Wu YH. Isotopic response of labetalol-associated lichen planus pemphigoides on an old radiation site: a case report. J Cutan Pathol. (2021) 48:1504–7.

# Sugawara A, Koga H, Abe T, Ishii N, Nakama T. Lichen planus-like lesion preceding bullous pemphigoid development after programmed cell death protein-1 inhibitor treatment. J Dermatol. (2021) 48:401–4.

# Yoshida S, Shiraishi K, Yatsuzuka K, Mori H, Koga H, Ishii N, et al. Lichen planus pemphigoides with antibodies against the BP180 C-terminal domain induced by pembrolizumab in a melanoma patient. J Dermatol. (2021) 48:e449–e451. doi: 10.1111/1346-8138.16006

# Boyle MM, Ashi S, Puiu T, Reimer D, Sokumbi O, Soltani K, et al. Lichen planus pemphigoides associated with PD-1 and PD-L1 inhibitors: a case series and review of the literature. Am J Dermatopathol. (2022) 44:360–7.

# Shah RR, Bhate C, Hernandez A, Ho CH. Lichen planus pemphigoides: A unique form of bullous and lichenoid eruptions secondary to nivolumab. Dermatol Ther. (2022) 35:15432. doi: 10.1111/dth.15432
